# Synthesis of new sulfonamides from sulfamethizole: in vitro antitubercular and antimicrobial activities supported by molecular docking, molecular dynamics, and ADME studies

**DOI:** 10.1007/s11030-026-11544-z

**Published:** 2026-04-12

**Authors:** Sevda Türk, Burak Kırılmaz, Elif Çiftçi, İsmail Çelik, Sevgi Karakuş, Dilek Şatana

**Affiliations:** 1 Department of Pharmaceutical Chemistry, Faculty of Pharmacy, Karadeniz Technical University, 61000 Trabzon, Turkey; 2 Department of Pharmaceutical Chemistry, Faculty of Pharmacy, Erciyes University, 38280 Kayseri, Turkey; 3 Department of Medical Microbiology, Faculty of Medicine, Istanbul Nisantasi University, 34398 Istanbul, Turkey; 4 Department of Pharmaceutical Chemistry, Faculty of Pharmacy, Istanbul Aydın University, 34295 Istanbul, Turkey; 5 Department of Medical Microbiology, Faculty of Medicine, Istanbul University, 34093 Istanbul, Turkey

**Keywords:** Sulfonamide, Antitubercular, Antimicrobial, Molecular docking, Molecular dynamics, ADME

## Abstract

The alarming increase in antimicrobial resistance and the persistence of diseases such as tuberculosis have created an urgent demand for novel agents with broad-spectrum antimicrobial potential. In this study, eleven new hybrid molecules (1–11) bearing sulfamethizole and benzamide scaffolds were synthesized using a molecular hybridization strategy. Structural elucidation was carried out via IR, ¹H-NMR, ¹³C-NMR, mass spectrometry, and elemental analysis. The synthesized compounds were evaluated for their biological activity against a diverse panel of microorganisms, including Gram-positive and Gram-negative bacteria, dermatophyte group mold-type fungi, yeast strains, and *Mycobacterium* species. Biological screening results revealed that several of the compounds exhibited notable antimicrobial activity, with certain derivatives showing enhanced efficacy against mycobacterial and fungal pathogens. Among them, compound 1 drew interest with its remarkable antifungal activity against *Trichophyton rubrum* and *Microsporum gypseum* (0.008 mM MIC for both), while compound 8 emerged as a potent antitubercular lead with a notable MIC of 0.031 mM against *Mycobacterium intracellulare*. Besides, according to the molecular docking studies performed on CYP51 enzyme, it showed the most promising binding in this group with a − 7.82 kcal/mol docking score, which is significantly better than the standard drug fluconazole (− 5.40 kcal/mol) and comparable to the co-crystallized ligand VT1 (− 9.36 kcal/mol), confirming its high binding affinity. Furthermore, 100 ns molecular dynamics simulations confirmed the structural stability of the enzyme-ligand complex through RMSD analysis, indicating a persistent binding mode. Complementary ADME predictions revealed that the compounds possess favorable pharmacokinetic properties and follow Lipinski’s Rule of Five. These findings highlight the potential of sulfamethizole-based benzamides as promising candidates for antimicrobial drug development through a successful correlation between in vitro and in silico data.

**Supplementary Information:**

The online version contains supplementary material available at 10.1007/s11030-026-11544-z.

## Introduction

Bacterial infections remain a significant global health challenge, accounting for millions of deaths each year. Although the discovery of antibiotics revolutionized modern medicine, the alarming rise in antibiotic-resistant bacterial strains has necessitated the search for new antimicrobial agents [1, 2]. Among infectious diseases, tuberculosis (TB) continues to be a major public health concern despite the availability of vaccines for over a century and the use of chemotherapeutic agents for more than seven decades. TB still contributes considerably to both morbidity and mortality worldwide [3].

Sulfonamides represent one of the earliest classes of synthetic antimicrobial agents and have been known for their broad-spectrum antibacterial activity since the 1930s [4]. The introduction of sulfa drugs marked a paradigm shift in the treatment of bacterial infections, significantly reducing mortality rates [5]. Since then, various sulfonamide derivatives with diverse biological properties have been developed and investigated. Recent studies have also demonstrated the potential of sulfonamides in other therapeutic areas, such as antitubercular [6], anticancer [7], antidiabetic [8], and carbonic anhydrase inhibitory activity [9]. Sulfamethizole, a clinically used sulfonamide, has been primarily employed in the treatment of urinary tract infections [10]. Moreover, sulfamethizole-based derivatives have shown promising antibacterial, anticancer, and carbonic anhydrase inhibition profiles in recent studies [11–13].

Benzamides are another class of compounds with a wide range of biological activities, including antitumor [14, 15], anti-Alzheimer [16], and antiviral effects [17]. Several benzamide-containing molecules have also demonstrated significant antimicrobial efficacy [18–21]. Given their diverse pharmacological potential, benzamide moieties are considered valuable scaffolds in drug discovery.

Molecular hybridization is an established strategy in medicinal chemistry, involving the combination of two or more pharmacophoric units within a single molecular framework to yield compounds with enhanced or multitarget biological activities [22, 23]. Inspired by the pharmacological significance of sulfonamides and benzamides, this study reports the design and synthesis of eleven novel sulfamethizole-benzamide hybrids. These compounds were synthesized via amidification between sulfamethizole and various benzoyl chloride derivatives, yielding structures in which the sulfamethizole core is connected to a benzamide linker. Such a modification enables a defined pharmacophoric arrangement that may facilitate interactions with biological targets related to mycobacterial and fungal strains. Following structural characterization by spectroscopic and elemental analyses, the resulting hybrids were comprehensively evaluated for their antimicrobial efficacy against a broad panel of clinically relevant bacterial and fungal pathogens.

## Experimental

### Chemistry

All of the chemicals, reagents and solvents were purchased from Sigma Aldrich (St. Louis, MO, USA) and Merck (Darmstadt, Germany). Melting points were determined by Schmelzpunktbestimmer SMP II apparatus. The IR spectra were recorded on a Schimadzu FTIR 8400 S Spectrometer. The NMR spectra were recorded (in DMSO-*d*_*6*_) with a Bruker spectrometer (Billerica, MA, USA) (300 MHz for ^1^H-NMR and 75 MHz for ^13^C-NMR, decoupled). The chemical shift values are expressed in ppm (δ scale) using tetramethylsilane as an internal standard. The mass spectral measurements were carried out by Electron Spray Ionization (ES) method on LC-MS-Agilent 1100. Elemental analysis was performed on Leco 215 CHNS-932 analyzer.

#### General procedure for the synthesis of compounds (1–11)

Sulfamethizole (0.003 mol) was dispersed in chloroform (20mL). Substitued benzoyl chlorides (0.003 mol) were also dispersed in chloroform (10mL) and added drop by drop onto Sulfamethizole. The reaction mixture was stirred at 25 °C for 1–3 h. The obtained precipitate was washed with water, dried and washed with hot ethanol to afford compounds 1–11 [24]. The synthesized compounds were monitored for reaction progress and purity using the TLC method. Fort his purpose silica gel plates (Merck F-254) with a thickness of 0.2 mm and dimensions of 20 × 20 cm were used as the adsorbent. For chromatographic analysis, a solvent system consisting of petroleum ether: ethyl acetate: glacial acetic acid (5:89:6, v/v) was employed. The spots corresponding to the compounds were detected as purple under a UV lamp at a wavelength of 254 nm.

***N*-{4-[(5-Methyl-1**,**3**,**4-thiadiazol-2-yl)sulfamoyl]phenyl}-4-nitrobenzamide (1)** Pale yellow solid; yield 67%; m.p.: 256–257 °C; Rf: 0.58; FT-IR: υ_max_ = 3356, 3219 (N–H), 3111 (=C–H), 2924, 2849 (C–H), 1686 (C=O), 1595, 1549, 1510 (C=C, N–H), 1344 (S=O), 831 (=C–H) cm^− 1^; ^1^H NMR (300 MHz, DMSO-*d*_*6*_): δ = 2.41 (s, 3 H, Ar–CH_3_), 7.71 (d, *J =* 8.4 Hz, 2 H, Ar–CH), 7.86 (d, *J =* 6.6 Hz, 2 H, Ar–CH), 8.09 (d, *J =* 6.6 Hz, 2 H, Ar–CH), 8.28 (d, *J =* 9.3 Hz, 2 H, Ar–CH), 10.79 (s, 1H, CONH), 13.84 (s, 1H, SO_2_NH) ppm; ^13^C NMR (75 MHz, DMSO-*d*_*6*_): δ = 168.2 (C=O); 164.7, 154.9, 149.7, 142.6, 140.4, 137.1, 129.7, 127.2, 124.00, 120.5 (Ar–C); 16.4 (CH_3_) ppm; EI-MS: 419 [M^+^]; Anal. Calcd. for C_16_H_13_N_5_O_5_S_2_ (419.43): C, 45.82; H, 3.12; N, 16.70; S, 15.29. Found: C, 45.75; H, 3.11; N, 16.67; S, 15.16.

***N*-{4-[(5-Methyl-1**,**3**,**4-thiadiazol-2-yl)sulfamoyl]phenyl}-4-chlorobenzamide (2)** White solid; yield 65%; m.p.: 269–271 °C; Rf: 0.56; FT-IR: υ_max_ = 3374, 3129 (N–H), 3042 (=C–H), 2911, 2805 (C–H), 1668 (C=O), 1591, 1522, 1487 (C=C, N–H), 1321 (S=O), 835 (=C–H) cm^− 1^; ^1^H NMR (300 MHz, DMSO-*d*_*6*_): δ = 2.59 (s, 3 H, Ar–CH_3_), 7.75 (d, *J =* 8.7 Hz, 2 H, Ar–CH), 7.91 (d, *J =* 9.3 Hz, 2 H, Ar–CH), 8.07 (d, *J =* 8.7 Hz, 2 H, Ar–CH), 8.12 (d, *J =* 8.7 Hz, 2 H, Ar–CH), 10.76 (s, 1H, CONH), 14.05 (bs, 1H, SO_2_NH) ppm; ^13^C NMR (75 MHz, DMSO-*d*_*6*_): δ = 168.4(C=O); 165.8, 165.0, 143.1, 137.4, 137.0, 133.7, 130.4, 129.1, 127.4, 120.6 (Ar–C); 16.7 (CH_3_) ppm; EI-MS: 408 [M^+^]; Anal. Calcd. for C_16_H_13_ClN_4_O_3_S_2_ (408.88): C, 47.00; H, 3.20; N, 13.70; S, 15.68. Found: C, 47.24; H, 3.28; N, 14.04; S, 15.58.

***N*-{4-[(5-Methyl-1**,**3**,**4-thiadiazol-2-yl)sulfamoyl]phenyl}-4-fluorobenzamide (3)** White solid; yield 69%; m.p.: 251–252 °C; Rf: 0.57; FT-IR: υ_max_ = 3352, 3187 (N–H), 3065 (=C–H), 2936, 2824 (C–H), 1655 (C=O), 1595, 1533, 1505, 1418 (C=C, N–H), 1327 (S=O), 1150 (S=O), 835 (=C–H) cm^− 1^; ^1^H NMR (300 MHz, DMSO-*d*_*6*_): δ = 2.47 (s, 3 H, Ar–CH_3_), 7.39 (t, 2 H, Ar–CH), 7.78 (d, *J =* 8.7 Hz, 2 H, Ar–CH), 7.94 (d, *J =* 8.7 Hz, 2 H, Ar–CH), 8.02–8.08 (m, 2 H, Ar–CH), 10.59 (s, 1H, CONH), 13.92 (bs, 1H, SO_2_NH) ppm; ^13^C NMR (75 MHz, DMSO-*d*_*6*_): δ = 168.3(C=O); 165.3, 164.7 (d, ^*1*^*J =* 248.0 Hz), 155.0, 143.1, 136.8, 131.3 (d, ^*4*^*J =* 2.5 Hz), 131.1 (d, ^*3*^*J =* 9.1 Hz), 127.3, 120.4, 115.9 (d, ^*2*^*J =* 21.8 Hz) (Ar–C); 16.5 (CH_3_) ppm; EI-MS: 393 [M^+^+1]; Anal. Calcd. for C_16_H_13_FN_4_O_3_S_2_ (392.43): C, 48.97; H, 3.34; N, 14.28;

S, 16.34. Found: C, 48.29; H, 3.31; N, 13.91; S, 16.21.

***N*-{4-[(5-Methyl-1**,**3**,**4-thiadiazol-2-yl)sulfamoyl]phenyl}-4-methylbenzamide (4)** White solid; yield 66%; m.p.: 270–271 °C; Rf: 0.52; FT-IR: υ_max_ = 3347, 3158 (N–H), 3057 (=C–H), 2926, 2824 (C–H), 1653 (C=O), 1611, 1591, 1532, 1508, 1418 (C=C, N–H), 1327 (S=O), 1146 (S=O), 826 (=C–H) cm^− 1^; ^1^H NMR (300 MHz, DMSO-*d*_*6*_): δ = 2.39 (s, 3 H, C_6_H_4_–CH_3_), 2.47 (s, 3 H, Ar–CH_3_), 7.35 (d, *J =* 8.1 Hz, 2 H, Ar–CH), 7.77 (d, *J =* 9.0 Hz, 2 H, Ar–CH), 7.88 (d, *J =* 8.4 Hz, 2 H, Ar–CH), 7.96 (d, J = 9.0 Hz, 2 H, Ar–CH), 10.49 (s, 1H, CONH), 13.92 (bs, 1H, SO_2_NH) ppm; ^13^C NMR (75 MHz, DMSO-*d*_*6*_): δ = 168.2 (C=O); 166.2, 154.9, 143.2, 142.5, 136.5, 131.9, 129.4, 128.2, 127.1, 120.3 (Ar–C); 21.4, 16.5 (CH_3_) ppm; EI-MS: 389 [M^+^+1]; Anal. Calcd. for C_17_H_16_N_4_O_3_S_2_ (388.46): C, 52.56; H, 4.15; N, 14.42; S, 16.51. Found: C, 51.61; H, 3.99; N, 14.25; S, 16.49.

***N*-{4-[(5-Methyl-1**,**3**,**4-thiadiazol-2-yl)sulfamoyl]phenyl}-4-methoxybenzamide (5)** White solid; yield 65%; m.p.: 262–264 °C; Rf: 0.48; FT-IR: υ_max_ = 3383, 3154 (N–H), 3057 (=C–H), 2934, 2820 (C–H), 1651 (C=O), 1611, 1591, 1530, 1505, 1416 (C=C, N–H), 1314 (S=O), 1136 (S=O), 835 (=C–H) cm^− 1^; ^1^H NMR (300 MHz, DMSO-*d*_*6*_): δ = 2.47 (s, 3 H, Ar–CH_3_), 3.85 (s, 3 H, OCH_3_), 7.08 (d, *J =* 9.0 Hz, 2 H, Ar–CH), 7.76 (d, *J =* 8.7 Hz, 2 H, Ar–CH), 7.93–7.99 (m, 4 H, Ar-CH), 10.42 (s, 1H, CONH), 13.92 (bs, 1H, SO_2_NH) ppm; ^13^C NMR (75 MHz, DMSO-*d*_*6*_): δ = 168.4 (C=O); 165.8, 162.8, 155.0, 143.5, 136.6, 130.4, 127.3, 127.0, 120.4, 114.3 (Ar–C); 56.1 (OCH_3_), 16.6 (CH_3_) ppm; EI-MS: 405 [M^+^+1]; Anal. Calcd. for C_17_H_16_N_4_O_4_S_2_ (404.46): C, 50.48; H, 3.99; N, 13.85; S, 15.86. Found: C, 49.94; H, 3.96; N, 13.78; S, 15.26.

***N*-{4-[(5-Methyl-1**,**3**,**4-thiadiazol-2-yl)sulfamoyl]phenyl}-4-bromobenzamide (6)** White solid; yield 71%; m.p.: 272–273 °C; Rf: 0.53; FT-IR: υ_max_ = 3374, 3123 (N–H), 3048 (=C–H), 2911, 2808 (C–H), 1667 (C=O), 1589, 1522, 1487, 1412 (C=C, N–H), 1323 (S=O), (=C–H) cm^− 1^; ^1^H NMR (300 MHz, DMSO-*d*_*6*_): δ = 2.47 (s, 3 H, Ar–CH_3_), 7.76–7.96 (m, 8 H, Ar–CH), 10.64 (s, 1H, CONH), 13.93 (bs, 0.8 H, SO_2_NH) ppm; ^13^C NMR (75 MHz, DMSO-*d*_*6*_): δ = 167.7 (C=O); 164.9, 154.4, 142.4, 136.3, 133.4, 131.4, 129.9, 126.7, 125.7, 119.9 (Ar–C); 16.0 (CH_3_) ppm; EI-MS: 453 [M^+^]; Anal. Calcd. for C_16_H_13_BrN_4_O_3_S_2_ (453.33): C, 42.39; H, 2.89; N, 12.36; S, 14.15. Found: C, 42.13; H, 2.94; N, 12.36; S, 13.22.

***N*-{4-[(5-Methyl-1**,**3**,**4-thiadiazol-2-yl)sulfamoyl]phenyl}-2**,**6-dichlorobenzamide (7)** White solid; yield 61%; m.p.: 258–259 °C; Rf: 0.54; FT-IR: υ_max_ = 3312, 3141 (N–H), 3032 (=C–H), 2974, 2876 (C–H), 1670 (C=O), 1591, 1526, 1431 (C=C, N–H), 1084, 839, 764 cm^− 1^; ^1^H NMR (300 MHz, DMSO-*d*_*6*_): δ = 2.47 (s, 3 H, Ar–CH_3_), 7.55–7.87 (m, 8 H, Ar–CH), 11.14 (s, 1H, CONH), 13.95 (bs, 0.9 H, SO_2_NH) ppm; ^13^C NMR (75 MHz, DMSO-*d*_*6*_): δ = 168.5 (C=O); 163.0, 155.1, 142.4, 137.4, 136.4, 132.2, 131.6, 128.8, 127.7, 119.8 (Ar–C); 16.6 (CH_3_) ppm; EI-MS: 443 [M^+^];. Anal. Calcd. for C_16_H_12_Cl_2_N_4_O_3_S_2_ (443.33): C, 43.35; H, 2.73; N, 12.64; S, 14.47. Found: C, 43.48; H, 2.97; N, 12.70; S, 14.58.

***N*-{4-[(5-Methyl-1**,**3**,**4-thiadiazol-2-yl)sulfamoyl]phenyl}-4-trifluoromethylbenzamide (8)** White solid; yield 71%; m.p.: 256–258 °C; Rf: 0.54; FT-IR: υ_max_ = 3356 (N–H), 3080 (=C–H), 2955, 2841 (C–H), 1658 (C=O), 1595, 1555, 1535, 1503, 1433 (C=C, N–H), 1317 (S=O), 827 (=C–H) cm^− 1^; ^1^H NMR (300 MHz, DMSO-*d*_*6*_): δ = 2.47 (s, 3 H, Ar–CH_3_), 7.78–8.17 (m, 8 H, Ar–CH), 10.79 (s, 1H, CONH), 13.94 (bs, 1H, SO_2_NH) ppm; ^13^C NMR (75 MHz, DMSO-*d*_*6*_): δ = 168.4 (C=O); 165.4, 155.1, 142.9, 138.8, 137.2, 129.3, 127.3, 126.0, 125.9, 120.6 (Ar–C); 16.6 (CH_3_) ppm; EI-MS: 443 [M^+^+1]; Anal. Calcd. for C_17_H_13_F_3_N_4_O_3_S_2_ (442.43): C, 46.15; H, 2.96; N, 12.66; S, 14.49. Found: C, 45.63; H, 3.22; N, 12.55; S, 14.39.

***N*-{4-[(5-Methyl-1**,**3**,**4-thiadiazol-2-yl)sulfamoyl]phenyl}-3-nitrobenzamide (9)** White solid; yield 75%; m.p.: 250–252 °C; Rf: 0.57; FT-IR: υ_max_ = 3273, 3152 (N–H), 3098 (=C–H), 2938, 2835(C–H), 1663 (C=O), 1648, 1595, 1533, 1495, 1422 (C=C, N–H), 1345, 1327 (S=O), 837 (=C–H) cm^− 1^; ^1^H NMR (300 MHz, DMSO-*d*_*6*_): δ = 2.47 (s, 3 H, Ar–CH_3_), 7.80–8.80 (m, 8 H, Ar–CH), 10.91 (s, 1H, CONH), 13.95 (bs, 1H, SO_2_NH) ppm; ^13^C NMR (75 MHz, DMSO-*d*_*6*_): δ = 168.4 (C=O); 164.4, 155.1, 148.3, 142.8, 137.3, 136.3, 134.9, 130.8, 127.4, 127.1, 123.1, 120.7 (Ar–C); 16.6 (CH_3_) ppm; EI-MS: 420 [M^+^+1]; Anal. Calcd. for C_16_H_13_N_5_O_5_S_2_ (419.43): C, 45.82; H, 3.12; N, 16.70; S, 15.29. Found: C, 45.56; H, 3.09; N, 16.40; S, 15.02.

***N*-{4-[(5-Methyl-1**,**3**,**4-thiadiazol-2-yl)sulfamoyl]phenyl}-3-chlorobenzamide (10)** White solid; yield 69%; m.p.: 245–247 °C; Rf: 0.61; FT-IR: υ_max_ = 3354, 3140 (N-H), 3036 (=C–H), 2911, 2807 (C–H), 1668 (C=O), 1589, 1522, 1497, 1474 (C=C, N–H), 1319 (S=O), 833 (=C–H) cm^− 1^; ^1^H NMR (300 MHz, DMSO-*d*_*6*_): δ = 2.47 (s, 3 H, Ar–CH_3_), 7.56–8.02 (m, 8 H, Ar–CH), 10.68 (s, 1H, CONH), 13.95 (bs, 0.8 H, SO_2_NH) ppm; ^13^C NMR (75 MHz, DMSO-*d*_*6*_): δ = 168.5 (C=O); 165.1, 155.1, 143.0, 137.1, 136.9, 133.8, 132.3, 131.1, 128.1, 127.4, 127.2, 120.6 (Ar–C); 16.7 (CH_3_) ppm; EI-MS: 409 [M^+^+1]; Anal. Calcd. for C_16_H_13_ClN_4_O_3_S_2_ (408.88): C, 47.00; H, 3.20; N, 13.70; S, 15.68. Found: C, 47.13; H, 3.28; N, 13.58; S, 15.22.

***N*-{4-[(5-Methyl-1**,**3**,**4-thiadiazol-2-yl)sulfamoyl]phenyl}-3-bromobenzamide (11)** White solid; yield 66%; m.p.: 223–225 °C; Rf: 0.65; FT-IR: υ_max_ = 3366, 3133 (N–H), 3051 (=C–H), 2903 (C–H), 1668 (C=O), 1589, 1533, 1516, 1472 (C=C, N–H), 1412, 1319 (S=O), 833 (=C–H) cm^− 1^; ^1^H NMR (300 MHz, DMSO-*d*_*6*_): δ = 2.47 (s, 3 H, Ar–CH_3_), 7.49–8.15 (m, 8 H, Ar–CH), 10.67 (s, 1H, CONH), 13.90 (bs, 0.8 H, SO_2_NH) ppm; ^13^C NMR (75 MHz, DMSO-*d*_*6*_): δ = 168.4 (C=O); 163.0, 155.1, 143.0, 137.2, 137.1, 135.3, 131.3, 131.0, 127.6, 127.4, 122.3, 120.6 (Ar–C); 16.7 (CH_3_) ppm; EI-MS: 453 [M^+^]; Anal. Calcd. for C_16_H_13_BrN_4_O_3_S_2_ (453.33): C, 42.39; H, 2.89; N, 12.36; S, 14.15. Found: C, 42.60; H, 3.04; N, 12.28; S, 13.16.

### Biological activity

#### Antitubercular activity assays

The antitubercular activity of the synthesized compounds was evaluated by the broth microdilution method that was performed according to the reference protocol of the Clinical and Laboratory Standards Institute (CLSI) [25–27]. *Mycobacterium tuberculosis* H37Rv ATCC 27,294 (American Type Culture Collection, USA) (*M. tuberculosis*; susceptible to all common antimycobacterial drugs), *M. tuberculosis* H37Rv ATCC 35,820 (resistant to streptomycin), *M. tuberculosis* H37Rv ATCC 35,822 (resistant to isoniazid), *M. tuberculosis* (one clinical isolate, resistant to isoniazid and rifampicin = multidrug resistant-MDR), *Mycobacterium intracellulare* ATCC 13,950 (*M. intracellulare)*, *Mycobacterium fourtuitum* ATCC 6841 (*M. fourtuitum*) and *Mycobacterium gordonae* (*M. gordonae;* one clinical isolate) were used in the experiments.

The final concentrations of the compounds were 1–0.0005 mM (12 dilutions). Stock solutions were obtained by preparing 40-fold dilutions of the final concentrations of the compounds in dimethyl sulfoxide (DMSO) and sterilized by filtration. Working stocks of 4 mM were prepared by diluting these solutions 1/10 in Middlebrook 7H9 broth medium (MB7H9) (Becton Dickinson, USA). Lyophilized powder form of isoniazid was used by diluting with sterile distilled water to a final concentration of 6–0.0012 µM [25–27]. The resazurin microtitre assay was used to determine the minimum inhibitory concentrations (MIC) according to the color changes at the end of incubation. Resazurin (Sigma-Aldrich, St. Louis, USA) was dissolved in sterile distilled water to a final concentration of 0.02%, sterilized by filtration, and stored at 4 °C until use [28]. *M. tuberculosis* isolates were subcultured onto Löwenstein-Jensen medium and incubated at 37 °C for 20–25 days. A few colonies were suspended in MB7H9 containing oleic acid-albumin-dextrose-catalase (OADC) to obtain 1.0 McFarland turbidity and followed by diluting 1:10 in MB7H9 [25–27].

The broth microdilution test was performed using sterile 96-well and U-shaped microdilution plates (LP Italiano SPA, Milano, Italy). Two microplates were used for each strain in the study. All of the wells contained 100µL MB7H9. Rows 1 to 12 contained serial dilutions of the compounds. In the first microplate, rows A–G, and in the second microplate, rows A–D, contained serial dilutions of compounds in a volume of 100 µL. In the second microplate, row E contained serial dilutions of isoniazid, row F contained DMSO control (1/10 DMSO/MB7H9), row G contained positive control (no drug), and row H contained negative control (broth only). Except for negative controls, 100 µL inoculum was added to each well. The microplates were incubated at 35 °C for about 7–14 days until mycobacterial growth (3–7 days for *M. fortuitum* and *M. gordonae*) was clearly visible as a white sediment in the positive control wells. Mycobacterial growth was confirmed by the Ehrlich–Ziehl–Neelsen acid-fast stain. The lowest concentrations of compounds that inhibited 100% of mycobacterial growth were defined as the MIC values. The MIC values were also confirmed by resazurin. Resazurin solution was added (30 µL) to each well, and the plates were incubated for one more day. The purple color was defined as negative, and the pink color was defined as positive for mycobacterial growth. The first purple-colored well was accepted as the MIC value [27, 28].

#### Antifungal activity assays

The microdilution method was used according to a standard protocol by the CLSI [29–32]. Nine strains were tested for each of the following species: *Microsporum gypseum* NCPF-580 (*M. gypseum*) (National Collection of Pathogenic Fungi, Public Health England), *Trichophyton mentagrophytes var. erinacei* NCPF 275 (*T. mentagrophytes var. erinacei*), *Trichophyton tonsurans* NCPF 245 (*T. tonsurans*), *Trichophyton simii* NCPF 392 (*T. simii*), *Trichophyton rubrum* (*T. rubrum;* clinical isolate ), *Candida parapsilosis* ATCC 22,019 (*C. parapsilosis)*, *Candida krusei* ATCC 6258 (*C. krusei*), *Candida albicans* ATCC 90,028 (*C. albicans*), *Candida auris* ATCC 8971 (*C. auris*) .

RPMI 1640 broth with L-glutamine without sodium bicarbonate and 0.165 M MOPS buffer (34.54 g/lt) were used. The medium was adjusted to pH 7.0 at 25 °C. Sterility control of each bottle was performed before it was used. Amphotericin B was provided by Sigma (catalog number: A4888) as standard powder, and fluconazole was provided by Sigma (catalog number: F8929) as standard powder. Amphotericin B, fluconazole, and compounds (1–11) were dissolved in 100% dimethyl sulfoxide recommended as CLSI guidelines [29–32]. The final concentrations of compounds (1–11) were 1 to 0.0005 mM, amphotericin B was 16 to 0.007 µg/mL, and fluconazole was 64 to 0.031 µg/mL.

Preparation of inoculum suspensions of dermatophytes was based on the CLSI guidelines [29]. The isolates were subcultured onto potato dextrose agar (PDA) plates at 30 °C for 4–5 days. The fungal colonies were covered with 1 ml of sterile 0.85% saline, and suspensions were made by gently probing the surface with the tip of a Pasteur pipette. The resulting mixture of conidia and hyphal fragments was withdrawn and transferred to a sterile tube. Heavy particles were allowed to settle for 5–10 min at room temperature; the upper suspension was mixed with a vortex for 15 s. The turbidity of supernatants was measured spectrophotometrically (Pharmacia, LKB. Ultrospec II) at a wavelength of 530 nm, and transmission was adjusted to 65 to 75%. These stock suspensions were diluted 1:50 in RPMI medium to obtain the final inoculum sizes, which range from 1 × 103 to 3 × 103 CFU/mL.

Preparation of inoculum suspensions of yeasts was based mainly on the CLSI guidelines [30–32]. The colonies of yeasts after 48 h at 35 °C of incubation onto Sabouraud dextrose agar (BBL, Sparks, MD, USA) were subcultured to 5 ml of sterile saline (0.85%) and turbidity was adjusted spectrophotometrically at 530 nm to 0.5 McFarland Standard, and firstly it was diluted 1:50 and then 1:20 in RPMI 1640 in order to obtain a final concentration of 0.5 × 103 to 2.5 × 103 CFU/ml.

The broth microdilution test was performed by using steril, disposable microdilution plates (96 U-shaped wells) (LP Italiano SPA, Milano, Italy). Two microplates were used for each strain in the study. All of the wells contained 100 µL RPMI 1640 medium. Rows 1 to 12 contained serial dilutions of the compounds. In the first microplate, rows A–G, and in the second microplate, rows A–D, contained serial dilutions of compounds in a volume of 100 µL. In the second microplate, row E contained serial dilutions of amphotericin B/fluconazole, row F contained DMSO control (1/10 DMSO/RPMI), row G contained positive control (no drug), and row H contained negative control (broth only). Each well was inoculated on the day of the test with 100 µl of the corresponding inoculum (except the negative control). This step brought the drug dilutions and inoculum size to the final test concentrations given above. The microplates that contained dermatophytes were incubated at 28 °C for 7 days. The microplates were read visually with the aid of an inverted reading mirror after 7 days for dermatophytes. For all drugs except fluconazole (80% inhibition), the minimal inhibitory concentrations (MICs) were defined as the lowest concentration of the drug (100% inhibition) that resulted in a complete inhibition of visible growth compared to that of drug-free growth control [29]. The MIC values were also confirmed by resazurin. Resazurin solution was added (30 µL) to each well, and the plates were incubated for one more day. The purple color was defined as negative, and the pink color was defined as positive for fungal growth. The first purple-colored well was accepted as the MIC value [32].

For yeasts, a constant volume (100 µl) of the inoculum was added to each microdilution well containing 100 µl of the serial dilution of drugs to reach final concentrations. The microplates were incubated at 35 °C for 48 h. The minimum concentration at which no growth was observed was taken as the MIC value, except for fluconazole (80% inhibition) [27–32]. The MIC values were also confirmed by resazurin. Resazurin solution was added (30 µL) to each well, and the plates were incubated for one more day. The purple color was defined as negative, and the pink color was defined as positive for fungal growth. The first purple-colored well was accepted as the MIC value [32].

#### Antibacterial activity assays

The microdilution method was used according to a standard protocol by the CLSI and the European Committee on Antimicrobial Susceptibility Testing (EUCAST) [33–37]. Seven strains were tested for each of the following species: *Bacillus cereus* ATCC 14,579 (*B. cereus)*, *Staphylococcus aureus* ATCC 29,213 (*S. aureus)*, *Staphylococcus epidermidis* ATCC 12,228 (*S. epidermidis)*, *Proteus vulgaris* ATCC 8427 (*P. vulgaris*), *Escherichia coli* ATCC 25,922 (*E. coli)*,* Klebsiella pneumoniae* ATCC 70,063 (*K. pneumoniae)*, and *Salmonella typhimurium* ATCC 14,028 (*S. typhimurium)*.

Mueller Hinton broth medium (MHB) (Becton and Dickinson, USA) was used for the microdilution method. The medium was adjusted to pH 7.0 at 25 °C. Sterility control of each bottle was performed before it was used.

Cefepime were provided by Sigma (catalog number: A3737) as standard lyophilized powders and dissolved in phosphate buffer 0.1 M (pH 6) solution. Compounds (1–11) were dissolved in 100% dimethyl sulfoxide recommended as CLSI guidelines [33]. The final concentrations of compounds (1–11) were 1 to 0.0005 mM, and cefepime was 64 to 0.031 µg/mL [33, 36].

Preparation of inoculum suspensions of bacteria was based mainly on the CLSI guidelines [33] and described previously [34]. The isolates were subcultured onto Mueller-Hinton agar medium at 37 °C for 24 h. A few colonies of freshly grown bacteria were suspended in 0.85% w/v sterile saline solution (OD600 = 0.063 in 0.85% w/v sterile saline) to obtain a final bacterial load of ~ 5 × 105 CFU/well.

The broth microdilution test was performed by using sterile, disposable microdilution plates (96 U-shaped wells) (LP Italiano SPA, Milano, Italy). Two microplates were used for each strain in the study. All of the wells contained 100 µL MHB. Rows 1 to 12 contained serial dilutions of the compounds. In the first microplate, rows A–G, and in the second microplate, rows A–D, contained serial dilutions of compounds in a volume of 100 µL. In the second microplate, row E contained serial dilutions of cefepime, row F contained DMSO control (1/10 DMSO/MHB), row G contained positive control (no drug), and row H contained negative control (broth only). The previously prepared inoculum was inoculated into each well as 10 µl. This step brought the drug dilutions and inoculum size to the final test concentrations given above. The microplates, which contained bacteria, were incubated at 37 °C until bacterial growth was clearly observed in the positive control row as white sediment (16–24 h). For all drugs, the minimal inhibitory concentrations (MICs) were defined as the lowest concentration of the drug that resulted in a complete inhibition of visible growth compared to that of drug-free growth control (100% inhibition) [33–37]. The MIC values were also confirmed by resazurin. Resazurin solution was added (30 µL) to each well. The purple color was defined as negative, and the pink color was defined as positive for bacterial growth. The first purple-colored well was accepted as the MIC value [34].

### In silico

#### Molecular docking study

In this study, the success of the compounds in the enzyme assay was the primary focus. The antifungal activities of the synthesized compounds (1–11) served as the starting point for determining the target structure in the docking study. Within this scope, compounds that could potentially be effective against the target sterol CYP51 [38] enzyme via molecular docking were analyzed. As in enzyme tests, fluconazole was used as the standard compound, and the co-crystal compound VT1 of the CYP51 enzyme’s (PDB ID: 5TZ1) crystal structure was also used for comparative analysis. Docking was performed using the Glide ligand docking module [39] in the Schrödinger Maestro interface. The three-dimensional structure of the target protein was obtained from the Protein Data Bank (PDB) and prepared using the Protein Preparation Wizard. Ligands were geometrically optimized using the LigPrep tool, and their protonation states were adjusted to pH 7.0 ± 0.2 conditions with OPLS4 force fields. Docking was performed using Glide’s standard precision (SP) modes. Docking results were analyzed and visualized using Discover Studio Visualizer v24, ProteinsPlus- Structure-Based Modeling Support Server, and PyMOL v3 Molecular Graphics System. The interaction energies and docking poses of the compounds were examined, and their interactions with the amino acid residues of the target enzyme were analyzed. To validate the molecular docking procedures, redocking was performed on the target protein’s co-crystal ligand, and the Root Mean Square Deviation (RMSD) [40] value was calculated.

#### Molecular dynamics simulation

In the molecular docking study, compound 1, the compound with the best interaction energy and docking pose, was subjected to molecular dynamics (MD) simulation to evaluate its dynamic properties and stability with the target enzyme. The input files for simulation were prepared using the CHARMM-GUI [41] server, selecting CHARMM36m force fields, and simulations were performed using Gromacs [42] v2023.3. The cubic simulation box was designed to leave a 10 Å buffer zone around the solute molecule, and the system was solvated using the TIP3P water model. To ensure electron neutrality, KCl ions at a concentration of 0.15 M were added, and molecular dynamics simulations were performed at a fixed temperature of 303 K and pressure of 1 atm. In the first step, an energy minimization process was applied to minimize steric clash and unwanted atomic interactions. Subsequently, a two-step equilibration protocol was followed: First, the system was equilibrated under fixed particle number, volume, and temperature conditions under the NVT ensemble; then, equilibration was continued under fixed particle number, pressure, and temperature values by switching to the NPT ensemble. After completing all preparation steps, the production phase was initiated, and a 100 ns molecular dynamics simulation was performed with 0.002 ps time steps. RMSD was used in the analysis of the simulation data.

#### ADME study

In silico ADME analyses were performed to evaluate the pharmacokinetic and toxicological profile of the compound 1, which emerged because of molecular docking and molecular dynamics simulations. SwissADME [43] an online platform for ADME predictions, was used in the study. The analyses examined the likelihood of gastrointestinal absorption, the potential to cross the blood-brain barrier, the plasma protein binding rate, the inhibitory or substrate properties on cytochrome P450 enzymes, metabolic stability, and various toxicity indicators. Lipinski’s five rules [44] were used as a reference in the evaluation of drug-like properties; the results were compared with the reference drug molecule fluconazole.

## Results and discussion

### Chemistry

**Fig. 1:**
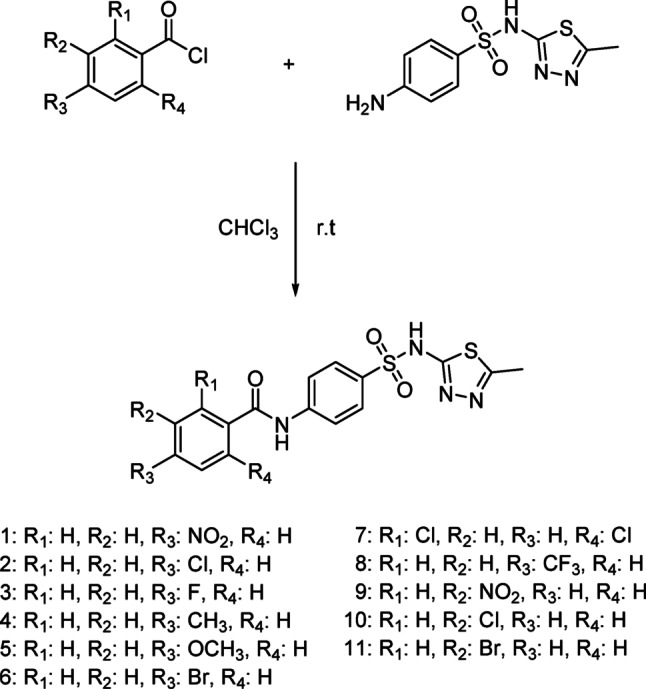
The synthetic route of compounds (1–11)

For the synthesis of target compounds 1–11, Sulfamethizole was reacted with various benzoyl chloride derivatives in chloroform media, at room temperature. The obtained product was purified by crystallization with ethanole. The synthetic route is given in Fig. 1.

The structures of the newly synthesized compounds (1–11) were characterized by elemental analysis and several spectroscopic methods (FT-IR, ^1^H-NMR, ^13^C-NMR, MS). According to the IR spectra, characteristic amide C=O stretching bands were obtained at 1686 –1651 cm^− 1^. Also, N–H stretching and aliphatic asymetric and symetric C–H stretching bands were determined at 3383 –3111 cm^− 1^ and 2974 − 2805 cm^− 1^, respectively. According to the ^1^H–NMR spectra, the methyl groups substituted to 1,3,4-thiadiazole ring were recorded as singlets between 2.41 and 2.59 ppm. The N–H protons assigned to amide and sulfonamide functional groups were appeared as singlets at 10.42–11.14 ppm and 13.84–14.05 ppm, respectively. In the ^13^C–NMR spectra, the amide C=O signals were recorded at 167.7–168.4 ppm. Distinctively, at compound 3 with fluoro substitution, four doublet peaks were recorded as *orto*,* meta*,* para* and *ipso* carbons in regard to fluor substituent. The calculated *J* values indicated carbons ^1^*J =* 248.0 Hz as *ipso*, ^2^*J =* 21.8 Hz as *ortho*, ^3^*J =* 9.1 Hz as *meta* and ^4^*J =* 2.5 Hz as *para* positions. Moreover, the MS spectra and elemental analysis results supported the compounds’ structure.

### Biological assay

#### Antitubercular, antifungal and antibacterial activities

The in vitro antitubercular activity of compounds 1–11 was tested against seven different *Mycobacterium* spp. strains: *M. tuberculosis* H37Rv ATCC 27,294 (susceptible to all common antimycobacterial drugs), *M. tuberculosis* H37Rv ATCC 35,820 (resistant to streptomycin), *M. tuberculosis* H37Rv ATCC 35,822 (resistant to isoniazid), *M. tuberculosis* (one clinical isolate and resistant to isoniazid and rifampicin = multidrug resistant-MDR), *M. intracellulare* ATCC 13,950, *M. fourtuitum* ATCC 6841, and *M. gordonae* (one clinical isolate). The clinical isolate was isolated from a sputum sample of a patient with tuberculosis and identified in the Mycobacteriology Laboratory of Istanbul Faculty of Medicine, Department of Medical Microbiology. The activity was expressed as minimum inhibitory concentration (MIC), the lowest concentration of compound that completely inhibited the growth on the culture, and determined by the broth microdilution method. As shown in Table 1, compounds 3, 5, 7, and 8 showed weak antitubercular activity against a range of *M. tuberculosis* strains with MIC values of 0.5 − 0.25 mM. However, test compounds 5 and 8 showed antitubercular activity below 0.125 mM against *M. intracellulare* (compound 8; 0.031 mM) and *M. gordonae* (compound 5; 0.125 mM).

**Table 1:** Antituberculosis activity investigation results of the synthesized compounds (1–11) against Mycobacterium species bacteria

	MIC Range: 1 – 0.0005 mM (12 dilutions)						
Code of the synthesized compounds	*M.tuberculosis* H37Rv ATCC 27294	*M.tuberculosis* ATCC 35820 Resistant to Streptomycin	*M.tuberculosis* ATCC 35822 resistant to INH	*M.tuberculosis* Resistant to INH+Rif (Clinical isolates)	*M.intracellulare* ATCC 13950	*M.fourtuitum* ATCC 6841	*M.gordonae* (Clinical isolates)
1	*1	>1	>1	1	>1	>1	1
2	>1	>1	>1	1	>1	>1	>1
3	0.5	1	0.5	0.5	0.5	1	0.5
4	1	>1	>1	1	1	>1	0.5
5	0.25	0.25	0.25	0.25	0.25	0.5	**0.125**
6	>1	>1	>1	1	>1	>1	>1
7	0.5	1	1	0.25	0.5	>1	0.5
8	1	1	0.5	1	**0.031**	1	0.5
9	1	1	1	1	0.25	>1	0.5
10	1	1	1	1	0.25	>1	1
11	1	1	1	1	>1	>1	1
DMSO control	+	+	+	+	+	+	+
Positive control	+	+	+	+	+	+	+
Negative control	−	−	−	−	−	−	−

*MIC

**Table 2:** Antifungal activity investigation results of the synthesized compounds (1–11) against dermatophyte group mold type fungi

Code of the synthesized compounds	MIC range: 1–0.0005 mM (12 dilutions)				
	T. simii NCPF 392	T.mentagrophytes var.erinacei NCPF 375	T. tonsurans NCPF 245	T.rubrum (Clinical strains)	M. gypseum NCPF 580
1	*0.062	0.031	0.125	**0.008**	**0.008**
2	0.5	0.062	0.25	0.062	0.031
3	> 1	0.062	0.25	0.062	0.062
4	> 1	0.031	0.25	0.062	0.25
5	> 1	0.031	0.25	0.125	0.5
6	> 1	0.062	0.25	0.125	0.25
7	> 1	0.062	0.5	0.25	0.5
8	0.062	0.062	0.125	0.031	**0.008**
9	0.25	0.031	0.25	0.062	**0.008**
10	> 1	0.062	0.25	0.062	**0.015**
11	> 1	0.062	0.25	0.062	0.031
Amphotericine B (16–0.007 µg/mL)	2 µg/mL	0.5 µg/mL	0.25 µg/mL	0.5 µg/mL	0.5 µg/mL
DMSO control	+	+	+	+	+
Positive control	+	+	+	+	+
Negative control	−	−	−	−	−

*MIC

As shown in Table 2, compounds 2, 3, 4, 5, 6, 7, 10, and 11 showed weak antifungal activity against *T. simii* NCPF 392, *T. tonsurans* NCPF 245 (compound 7), and *M. gypseum NCPF 580 (*compounds 5 and 7*) strains* with MIC values of *≥* 1–0.5 mM. The test compounds 1, 8, 9, and 10 showed good antifungal activity against T. *rubrum* and *M. gypseum NCPF 580* strains with MIC values of 0.008–0.015 mM.

**Table 3:** Antifungal activity investigation results of the synthesized compounds (1–11) against yeasts

Code of the synthesized compounds	MIC Range: 1–0.0005 mM (12 dilutions)			
	C. albicans ATCC 90,028	C. auris NCPF 8971	C. krusei ATCC 6258	C. parapsilosis ATCC 22,019
1	> 1	> 1	> 1	> 1
2	> 1	> 1	> 1	> 1
3	> 1	> 1	> 1	> 1
4	> 1	> 1	> 1	> 1
5	> 1	> 1	> 1	> 1
6	> 1	> 1	> 1	> 1
7	> 1	> 1	> 1	> 1
8	> 1	> 1	> 1	> 1
9	> 1	> 1	> 1	> 1
10	> 1	> 1	> 1	> 1
11	> 1	> 1	> 1	> 1
Fluconazole (64–0.031 µg/mL)	0.125 µg/mL	32 µg/mL	8 µg/mL	32 µg/mL
Amphotericine B (16–0.007 µg/mL)	0.125 µg/mL	0.5 µg/mL	0.5 µg/mL	0.5 µg/mL
DMSO control	+	+	+	+
Positive control	+	+	+	+
Negative control	−	−	−	−

*MIC

As shown in Table 3, all compounds showed weak antifungal activity against *the yeast* with MIC values of *≥* 1 mM.

**Table 4:** Antibacterial activity investigation results of the synthesized compounds (1–11) against Gram positive bacteria

Code of the synthesized compounds	MIC Range: 1–0.0005 mM (12 dilutions)		
	Bacillus cereus ATCC 14,579	Staphylococcus aureus ATCC 29,213	Staphylococcus epidermidis ATCC 12,228
1	*>1	> 1	**0.25**
2	> 1	> 1	1
3	> 1	> 1	1
4	> 1	> 1	1
5	1	> 1	1
6	1	1	1
7	> 1	> 1	**0.5**
8	1	> 1	> 1
9	> 1	> 1	> 1
10	> 1	> 1	> 1
11	1	1	1
Cefepime (64–0.031 µg/mL)	2 µg/mL	1 µg/mL	1 µg/mL
DMSO control	+	+	+
Positive control	+	+	+
Negative control	−	−	−

*MIC

**Table 5:** Antibacterial activity investigation results of the synthesized compounds (1–11) against Gram negative bacteria

Code of the synthesized compounds	MIC Range: 1–0.0005 mM (12 dilutions)			
	Proteus vulgaris ATCC 8427	Escherichia coli ATCC 25,922	Klebsiella pneumoniae ATCC 70,063	Salmonella typhimurium ATCC 14,028
1	*1	> 1	> 1	> 1
2	> 1	> 1	> 1	> 1
3	1	**0.25**	> 1	> 1
4	1	> 1	> 1	> 1
5	> 1	> 1	> 1	> 1
6	**0.25**	> 1	> 1	> 1
7	> 1	> 1	> 1	> 1
8	> 1	> 1	> 1	> 1
9	> 1	> 1	> 1	> 1
10	> 1	> 1	> 1	> 1
11	> 1	> 1	> 1	> 1
Cefepime (64–0.031 µg/mL)	1 µg/mL	0.125 µg/mL	32 µg/mL	2 µg/mL
DMSO control	+	+	+	+
Positive control	+	+	+	+
Negative control	−	−	−	−

*MIC

As shown in Tables 4 and 5, all compounds showed weak antibacterial activity against the bacteria with MIC values of *≥* 1 mM (except for compounds 1, 3, 6, and 7).

For antimicrobial activity study, the experimental conditions were repeated three times, and consistent results were obtained. The obtained results have been presented in Tables 1, 2, 3, 4 and 5. The compounds demonstrating significant activity have been highlighted in bold.

### In silico study

#### Molecular docking study

To validate the reliability of the molecular docking protocol, a redocking procedure was performed using the co-crystallized ligand (VT1) of CYP51 (PDB ID: 5TZ1). The root mean square deviation (RMSD) between the docked pose and the crystallographic conformation was calculated and found to be 0.4 Å, indicating that the docking protocol accurately reproduces the experimentally observed binding mode. The analysis revealed that compound 1 exhibited stronger binding energies and stable interaction networks compared to the other compounds examined in this study. Compound 1 has an interaction score of − 7.82 kcal/mol with the CYP51 protein. Compound 1 exhibited a higher binding affinity than the reference drug fluconazole, with a binding energy of − 7.82 kcal/mol, and demonstrated strong binding potential by effectively mimicking the key binding sites of the high-affinity co-crystal ligand VT1. The docking interaction energies and glide e-model scores of the compounds, the co-crystal VT1, and the standard compound fluconazole are provided in Table 6.

**Table 6:** Docking interaction energies and Glide emodel scores of compounds 1–11, co-crystal ligand VT1, and fluconazole

Compound	Docking score (kcal/mol)	Glide emodel (kcal/mol)	H-Bonds	Hydrophobic interactions
1	− **7.82**	− **76.826**	–	Tyr118, His377, Phe233, Leu88, Met508
2	− 7.71	− 72.026	–	Tyr64, Tyr118, His377, Phe233, Pro230, Leu88, Met508
3	− 7.71	− 69.668	–	Tyr64, Tyr118, His377, Phe233, Pro230, Leu87, Leu376, Met508
4	− 7.72	− 71.933	–	Tyr64, His377, Phe233, Leu87, Leu88
5	− 7.63	− 73.213	–	Tyr64, His377, Phe233, Leu87, Leu88, Ser378
6	− 7.47	− 73.347	His377, Met508	Tyr64, Tyr118, Phe126, Phe233, Pro230, Leu87, Leu88, Leu376
7	− 6.50	− 55.234	–	Tyr64, Pro230, Gly65, Leu87, Leu376, Ser378
8	− 7.76	− 72.852	His377, Met508	Tyr64, Tyr118, Phe233, Pro230, Leu87, Leu88, Leu376
9	− 5.75	− 84.772	–	Tyr64, Tyr118, His377, Phe233, Pro230, Leu88, Leu376, Ser506
10	− 7.81	− 74.265	–	Tyr64, Tyr118, His377, Phe233, Pro230, Gly65, Leu87, Leu376
11	− 7.61	− 76.729	–	Tyr64, Tyr118, His377, Phe233, Pro230, Gly65, Leu87, Leu88, Leu376
VT1	− 9.36	− 94.119	–	Tyr118, His377, Phe233, Pro230, Gly303, Met508, Hem-601
Fluconazole	− 5.40	− 56.848	–	Tyr118, Cys470, Gly303, Hem-601

**Fig. 2:**
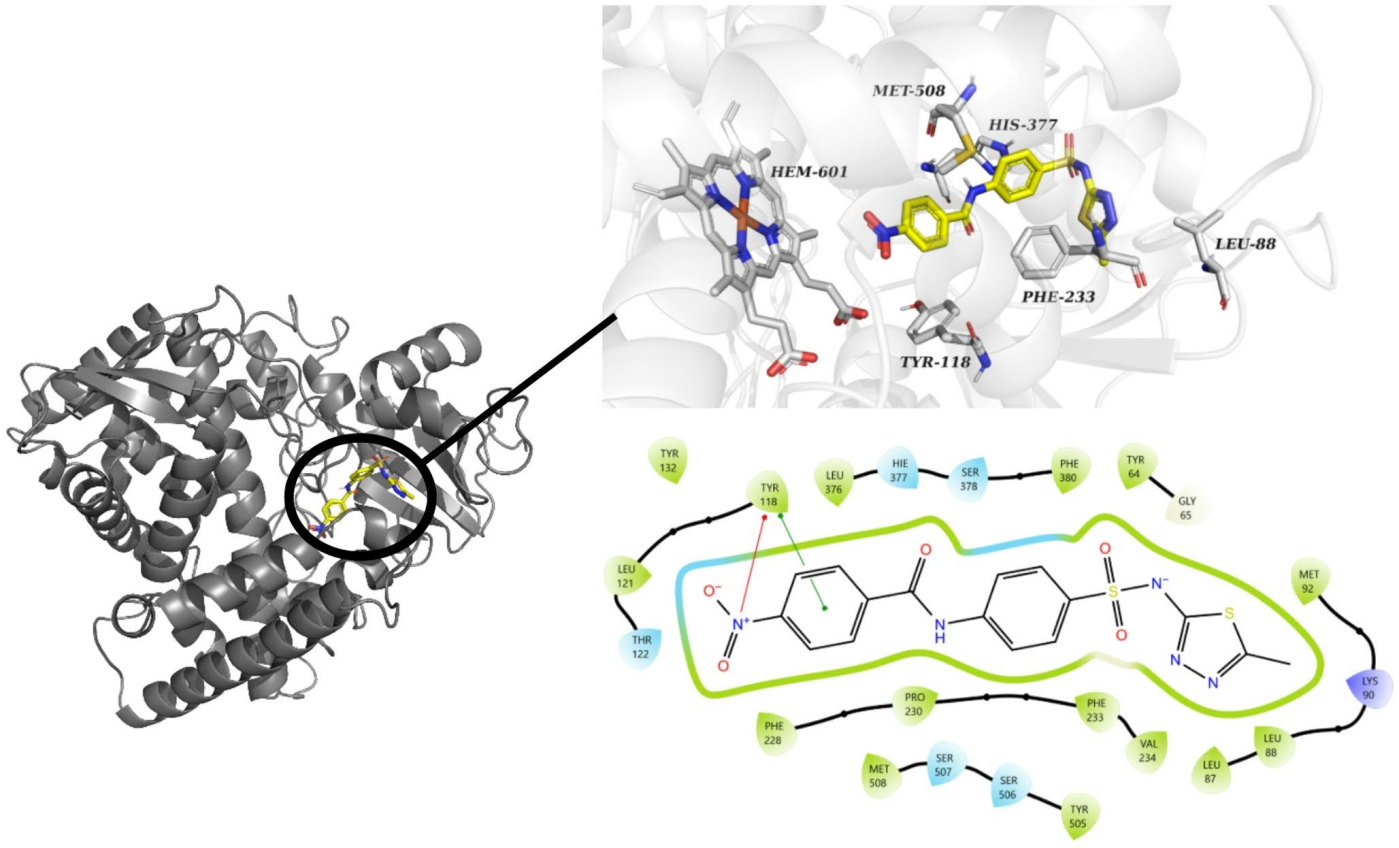
Binding pose and interaction diagram of active compound 1 on the CYP51 active site (PDB: 5TZ1)

Although compound 1 exhibited a favorable docking score compared to fluconazole, it should be noted that docking scores represent relative ranking rather than absolute binding affinities. The stronger docking score of the co-crystallized ligand VT1 is consistent with its experimentally confirmed binding mode and supports the validity of the docking protocol. Compound 1 forms π-π cation interactions with the substituted nitro group at Tyr118 and π–π stacking hydrophobic interactions with the phenyl ring to which it is attached during its interaction with the CYP51 protein. Furthermore, the hydrophobic bonds it forms with the amino acid residues His377 and Phe233 significantly strengthen the compound’s position in the active site. Furthermore, it forms hydrophobic interactions with Leu88 and Met508. Notably, this interaction pattern shows significant similarities with the standard antifungal fluconazole, which also relies on interactions with Tyr118 and the surrounding hydrophobic environment to exert its inhibitory effect. Similarly, the co-crystal ligand VT1 also exhibits hydrophobic interactions with His377, Phe233, and Met508, binding strongly to the active site. This high degree of conservation in the binding mode, particularly with these key residues, suggests that compound 1 effectively occupies the catalytic niche in a manner similar to VT1, justifying its superior docking performance and high docking score. The binding positions of compound 1 are shown in Fig. 2.

#### Molecular dynamics simulation

**Fig. 3:**
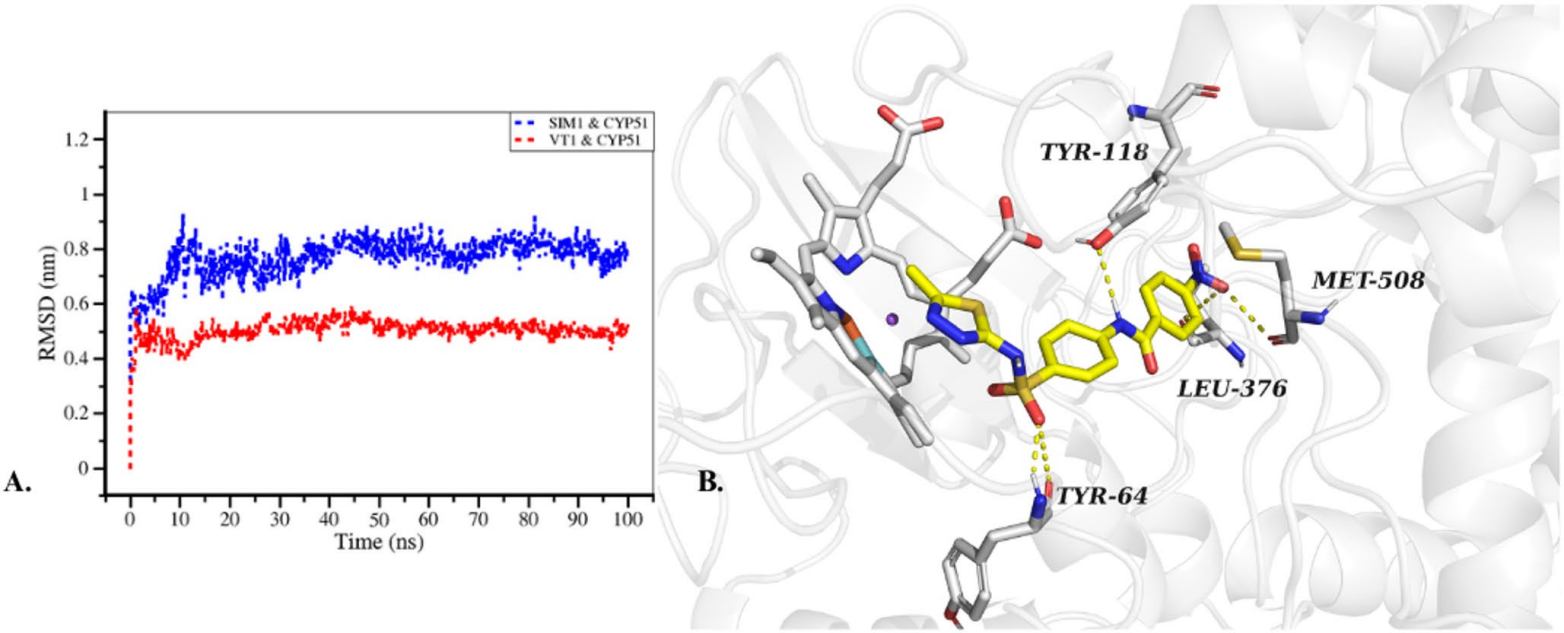
**A** RMSD changes in the compound 1–CYP51 and VTI–CYP51 complexes throughout the simulation. **B** Binding mode and interacting amino acid residues in the active site of CYP51 at 100 ns for compound 1

To investigate the stability of the compound 1 & CYP51 protein-ligand complex obtained from the Glide SP molecular docking study, 100 ns MD simulations were performed. Additionally, under the same conditions, the co-crystal ligand-protein, VT1 & CYP51 complex, was included in the MD simulation as a control group. The results were interpreted by analyzing the trajectory coordinate file recorded as a frame in the MD simulations, and RMSD analyses were performed for each complex (Fig. 3).

RMSD measurements provide fundamental information about the time-dependent shifts and deviations of the protein and ligand. As seen in Fig. 3A, the compound 1 & CYP51 complex was below 0.7 nm in the first 10 ns and stabilized around 0.8 nm from 10 ns to 100 ns. The plateau observed in the RMSD profile following the initial equilibration phase indicates that the enzyme-ligand complex has reached a state of structural stability. This consistency observed over the 100 ns trajectory demonstrates that compound 1 remains firmly anchored within the active site of CYP51 and does not induce significant conformational changes in the protein backbone. Although some hydrophobic bonds formed by compound 1 broke by the 10th ns, it increased its binding affinity by forming hydrogen bonds with the amino acid residues Tyr118, Tyr64, Leu376, Ser63, and Met508. Additionally, it was observed to form a coordination bond with the HEME group in the 10–100 ns range. The VT1 & CYP51 complex stabilized around 0.5 nm throughout the simulation, although it decreased to around 0.4 nm at 10 ns. The binding pose of compound compound 1 at 100 ns is shown in Fig. 3B.

Binding scores reflect relative ligand ranking rather than absolute binding affinities, as solvent effects, entropic contributions, and long-timescale protein dynamics cannot be fully captured. Therefore, binding and molecular dynamics results provide a computational framework to support compound prioritization and should be interpreted in conjunction with experimental biological activity data.

#### ADMET study

**Fig. 4:**
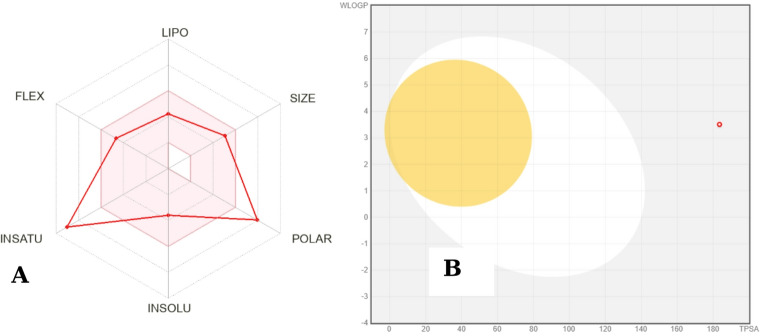
**A** Radar chart of compound 1 expressing physicochemical properties obtained from SwissADME server. **B** BOILED-Egg diagram showing ADME properties such as GI absorption and BBB permeant

Physicochemical property calculations for compound compound 1 showed that the compound has a molecular weight of 419.43 g/mol, 7 hydrogen bond acceptors, 2 hydrogen bond donors, 7 rotatable bonds, a Csp3 fraction of 0.06, and a TPSA value of 183.49 Å². The calculated consensus Log Po/w value for lipophilicity was found to be 1.37. Water solubility calculations showed complete, moderate, and moderate solubility, respectively, according to the Log S (ESOL) (− 3.62), Log S (Ali) (− 5.37), and Log S (SILICOS-IT) (− 5.61) values. The radar map showing the physicochemical parameters of compound 1 is shown in Fig. 4A. The yellow middle part of the BOILED-Egg graph indicates that the compound can cross the BBB via passive diffusion, the white area indicates GI absorption, and the red dot indicates that the compound cannot be excreted from the central nervous system by P-glycoprotein. As shown in the BOILED-Egg diagram in Fig. 4B, compound 1 has low GI absorption, does not cross the BBB, and does not function as a P-gp substrate. It exhibited inhibitory properties for CYP2C9, CYP2C19, and CYP3A4. The calculated limiting rules for drug-like values were consistent with Lipinski (0 violations) and Ghose but not with Veber, Egan, and Muegge’s limiting rule (1 violation: above TPSA limits).

## Conclusion

In this study, eleven novel sulfamethizole-benzamide hybrids were synthesized and evaluated for their broad-spectrum antimicrobial potential. Biological assays identified compound 1 and compound 8 as the most significant leads, exhibiting remarkable antifungal activity against *T. rubrum* and *M. gypseum* (MIC: 0.008 mM) and notable antitubercular potency against M. intracellulare (MIC: 0.031 mM). The remarkable antifungal potency of compound 1 and the significant anti-mycobacterial profile of compound 8 identify them as promising leads, validating the biological efficacy of the synthesized sulfamethizole-benzamide scaffold. Structure-activity relationship observations suggest that halogen and nitro substitutions on the benzamide moiety enhance antimicrobial efficacy. Computational studies further validated these results, as compound 1 demonstrated high binding affinity and structural stability within the CYP51 active site, supported by a favorable pharmacokinetic profile. These findings position the synthesized hybrids as promising precursors for the development of potent new antimicrobial and antitubercular agents.

## Supplementary Information

Below is the link to the electronic supplementary material.

Supplementary Material 1

## Data Availability

No datasets were generated or analysed during the current study.
