# Supplementary material for: Synthesis of new sulfonamides from sulfamethizole: in vitro antitubercular and antimicrobial activities supported by molecular docking, molecular dynamics, and ADME studies
Source: Mol Divers. 2026 Apr 12;30(4):6389–402. doi: 10.1007/s11030-026-11544-z (PMC13332992; doi:10.1007/s11030-026-11544-z)
Supplement: Supplementary file 1 — Supplementary Material 1 [file 11030_2026_11544_MOESM1_ESM.docx]

**SUPPLEMENTARY DATA**

**Title:** Design and synthesis of novel sulfamethizole-based benzamides as potential antibacterial, antifungal, and antimycobacterial agents

Sevda Türk^a*^, Burak Kırılmaz^b^, Elif Çiftçi^c^, İsmail Çelik^b^, Sevgi Karakuş^d^, Dilek Şatana^e^

^a^Department of Pharmaceutical Chemistry, Faculty of Pharmacy, Karadeniz Technical University, 61000, Trabzon, Türkiye

^b^Department of Pharmaceutical Chemistry, Faculty of Pharmacy, Erciyes University, 38280, Kayseri, Türkiye

^c^Department of Medical Microbiology, Faculty of Medicine, Istanbul Nisantasi University, 34398, Istanbul, Türkiye

^d^Department of Pharmaceutical Chemistry, Faculty of Pharmacy, Istanbul Aydın University, 34295, Istanbul, Türkiye

^e^Department of Medical Microbiology, Faculty of Medicine, Istanbul University, 34093, Istanbul, Türkiye

***Correspondence Author:**

Sevda TÜRK

Department of Pharmaceutical Chemistry, Faculty of Pharmacy, Karadeniz Technical University, Trabzon, Türkiye

Phone number: +90 462 325 6762

Fax number: +90 462 325 6717

E-mail: [sevdaturk@ktu.edu.tr](mailto:sevdaturk@ktu.edu.tr)

**
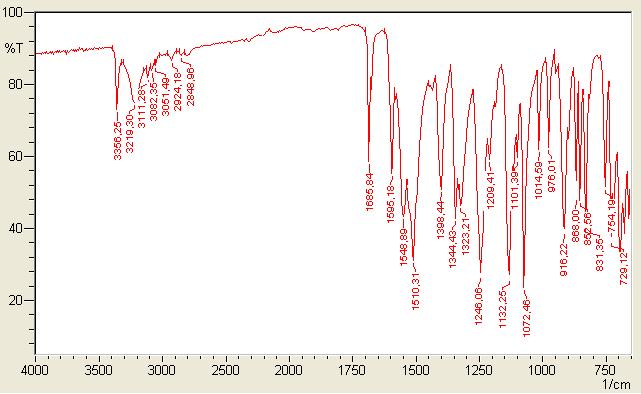
**

**Figure S1.** FTIR spectrum of compound **1**


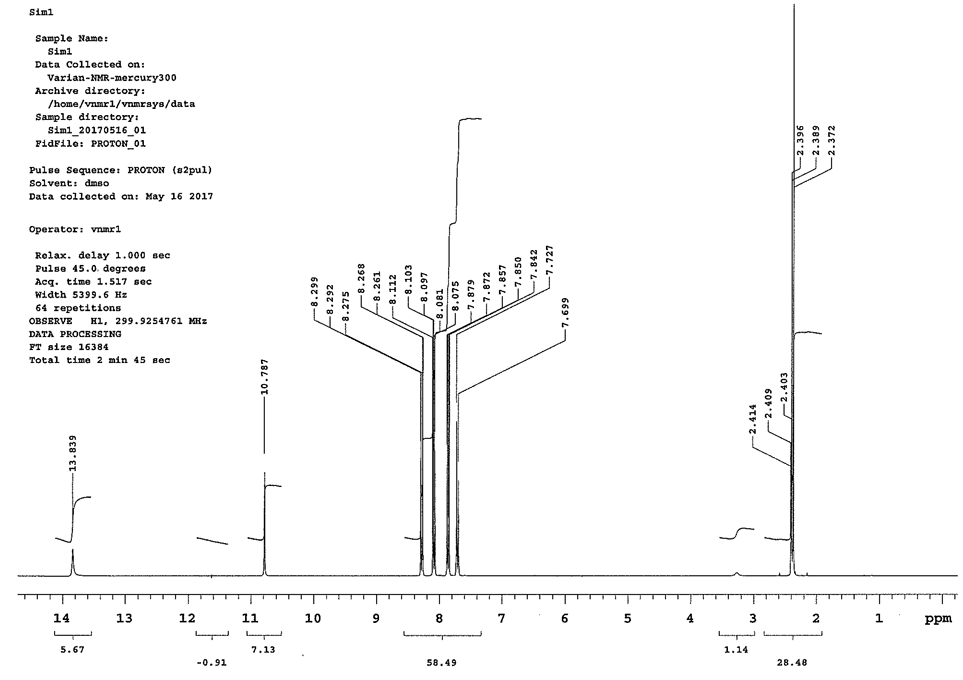


**Figure S2.** ^1^H-NMR spectrum of compound **1**


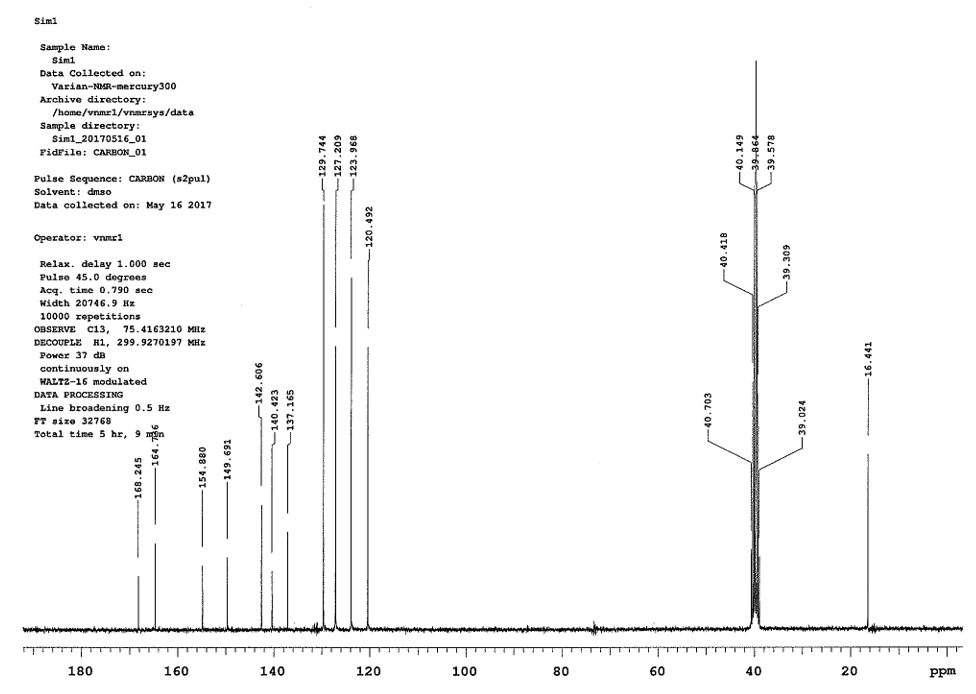


**Figure S3.** ^13^C-NMR spectrum of compound **1**


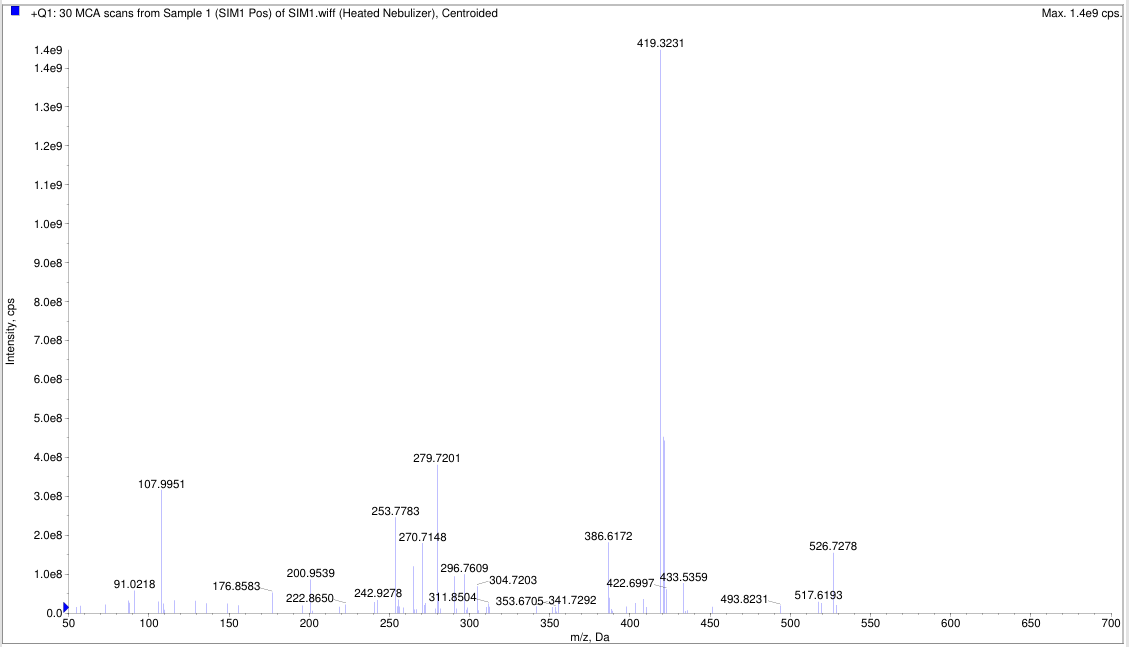


**Figure S4.** Mass spectrum of compound **1**

**
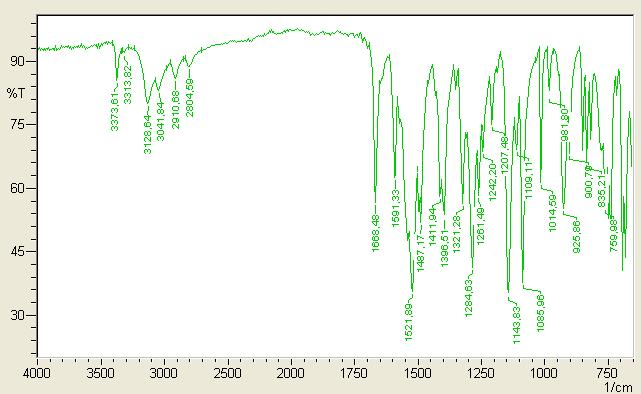
**

**Figure S5.** FTIR spectrum of compound **2**


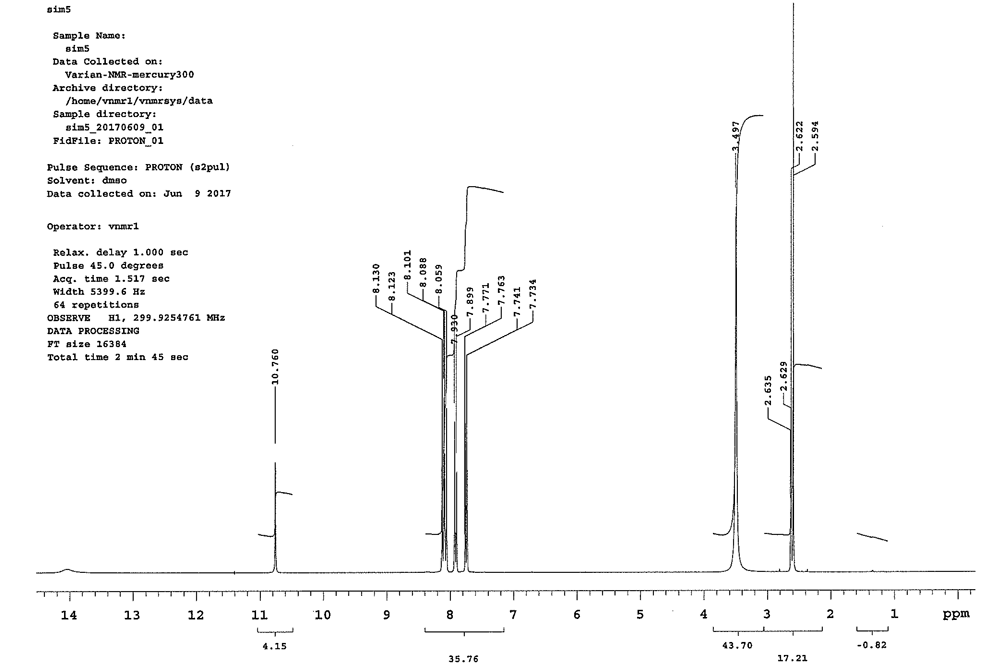


**Figure S6.** ^1^H-NMR spectrum of compound **2**


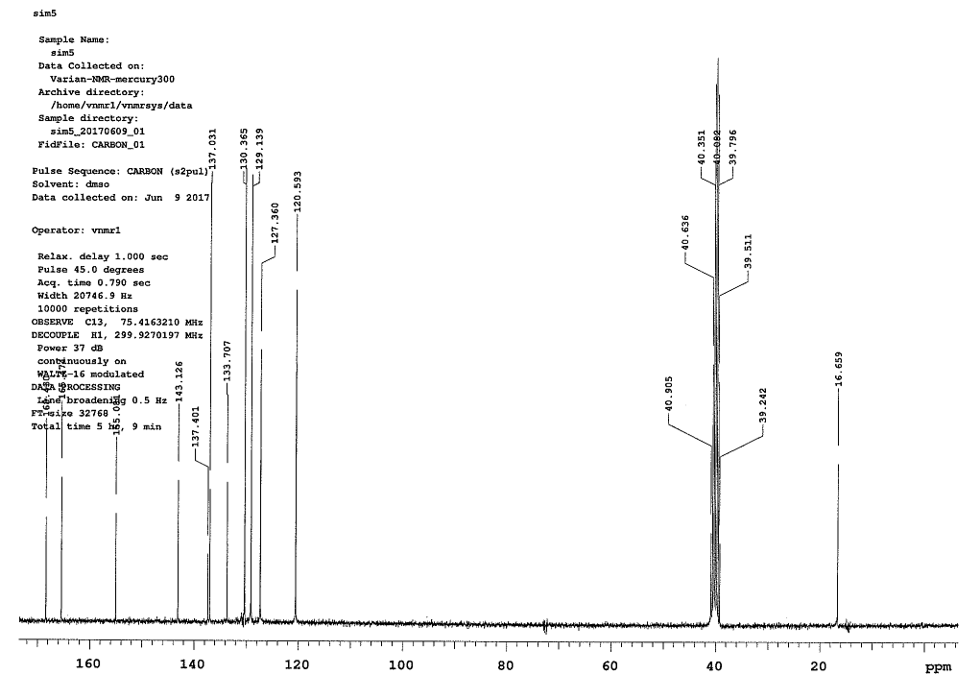


**Figure S7.** ^13^C-NMR spectrum of compound **2**


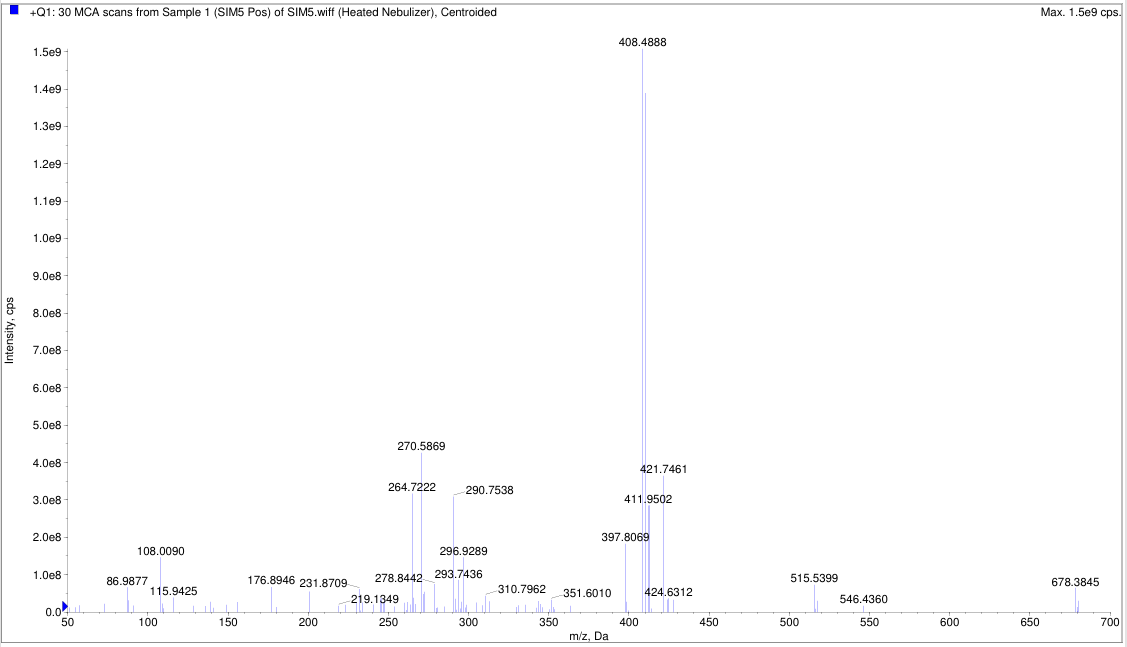


**Figure S8.** Mass spectrum of compound **2**

**
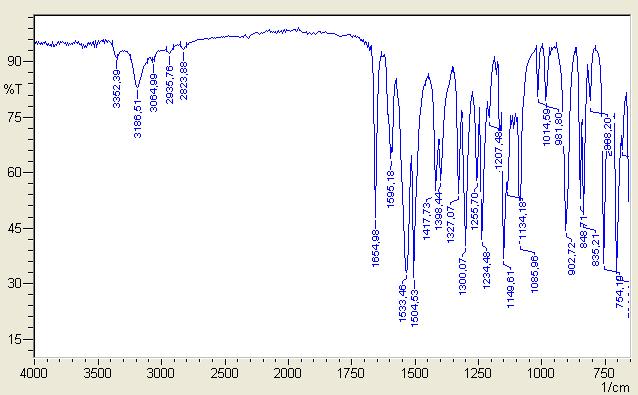
**

**Figure S9.** FTIR spectrum of compound **3**

**
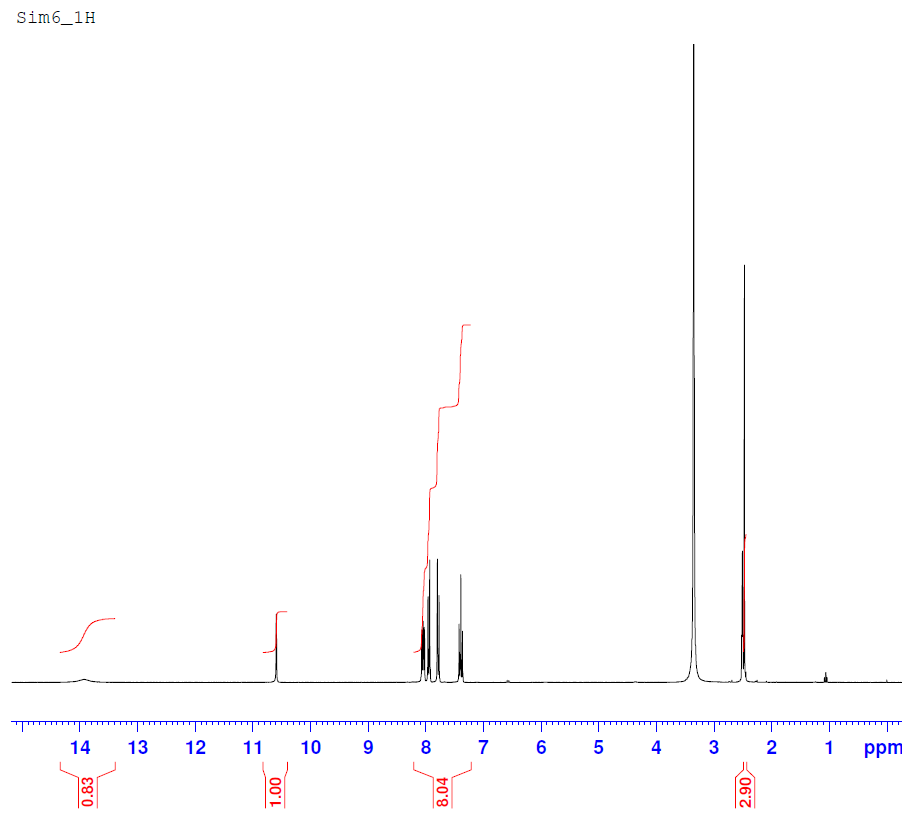
**

**Figure S10.** ^1^H-NMR spectrum of compound **3**


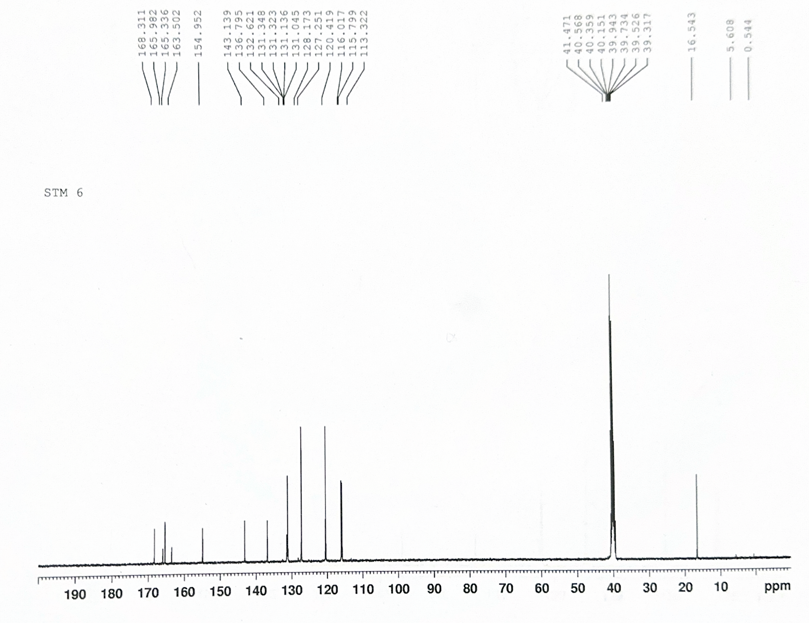


**Figure S11.** ^13^C-NMR spectrum of compound **3**


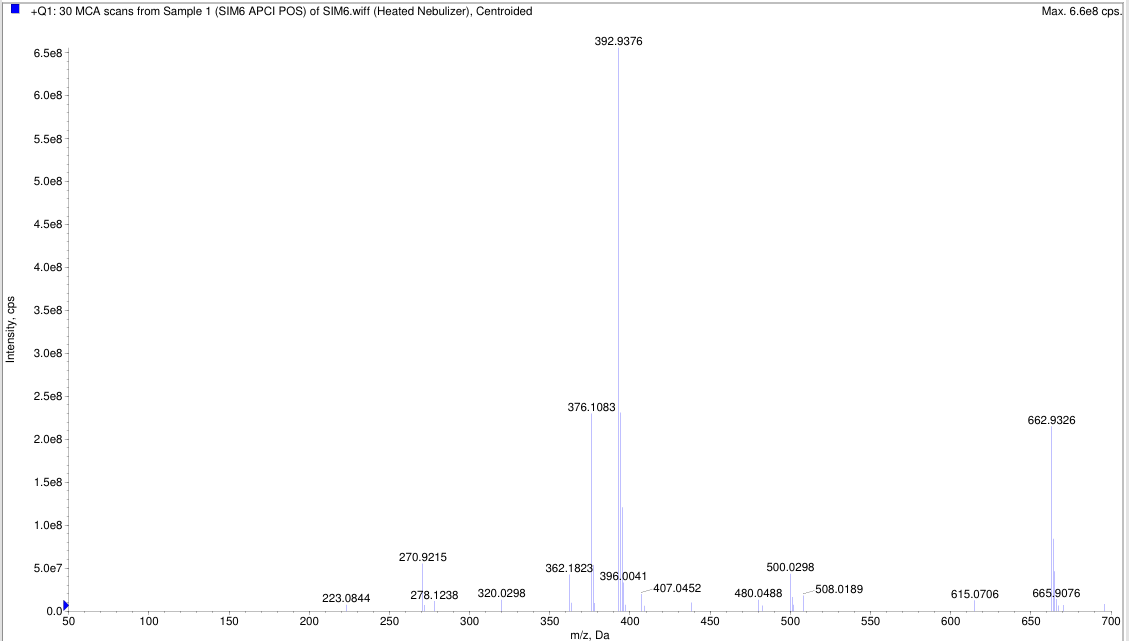


**Figure S12.** Mass spectrum of compound **3**

**
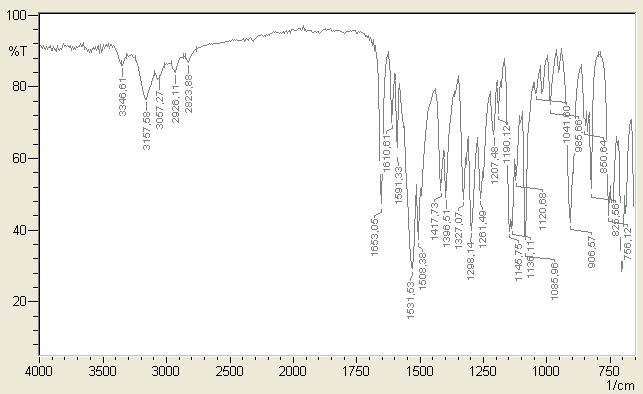
**

**Figure S13.** FTIR spectrum of compound **4**

**
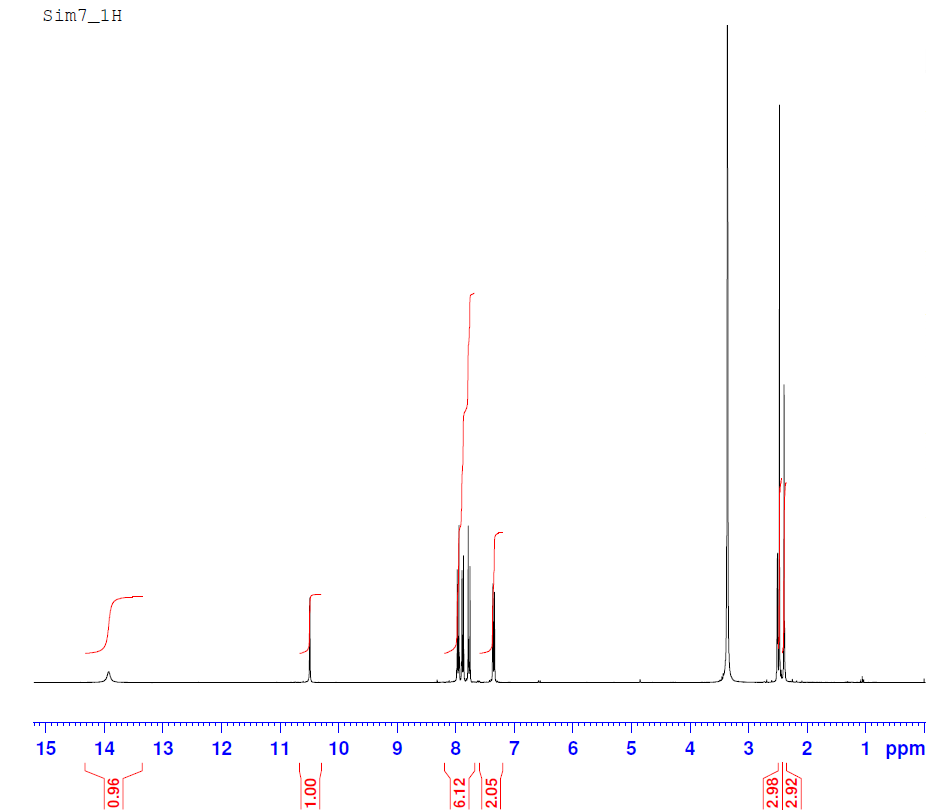
**

**Figure S14.** ^1^H-NMR spectrum of compound **4**


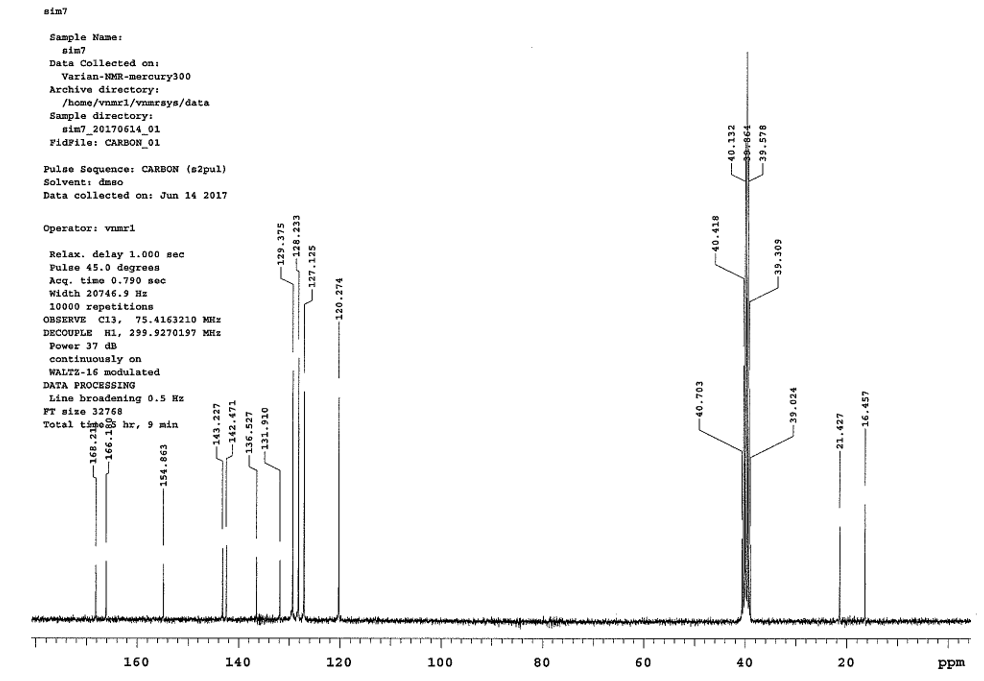


**Figure S15.** ^13^C-NMR spectrum of compound **4**


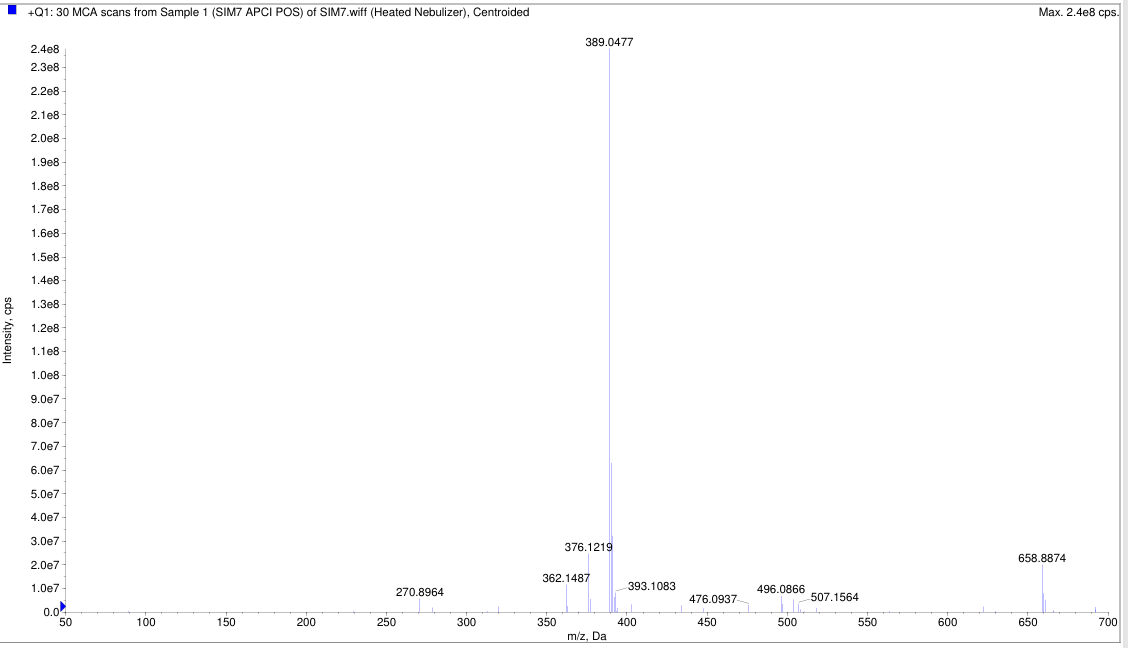


**Figure S16.** Mass spectrum of compound **4**


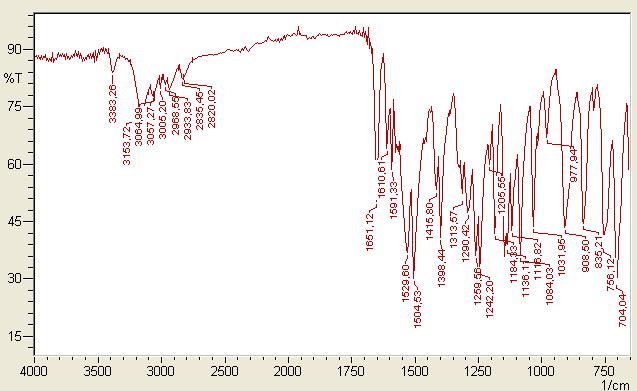


**Figure S17.** FTIR spectrum of compound **5**


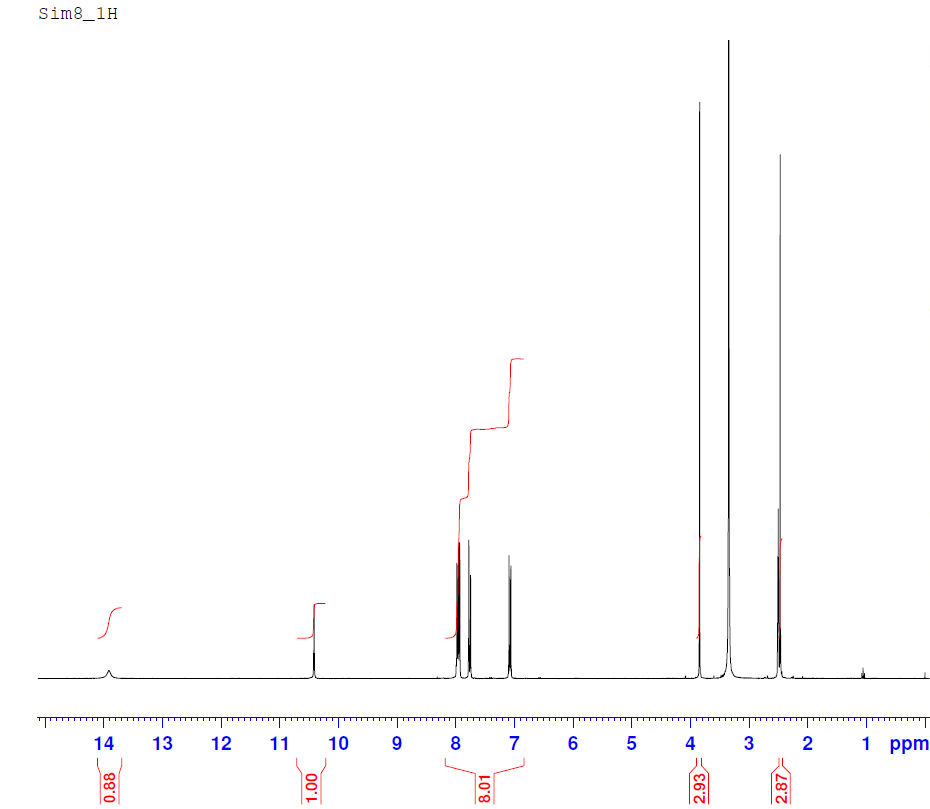


**Figure S18.** ^1^H-NMR spectrum of compound **5**


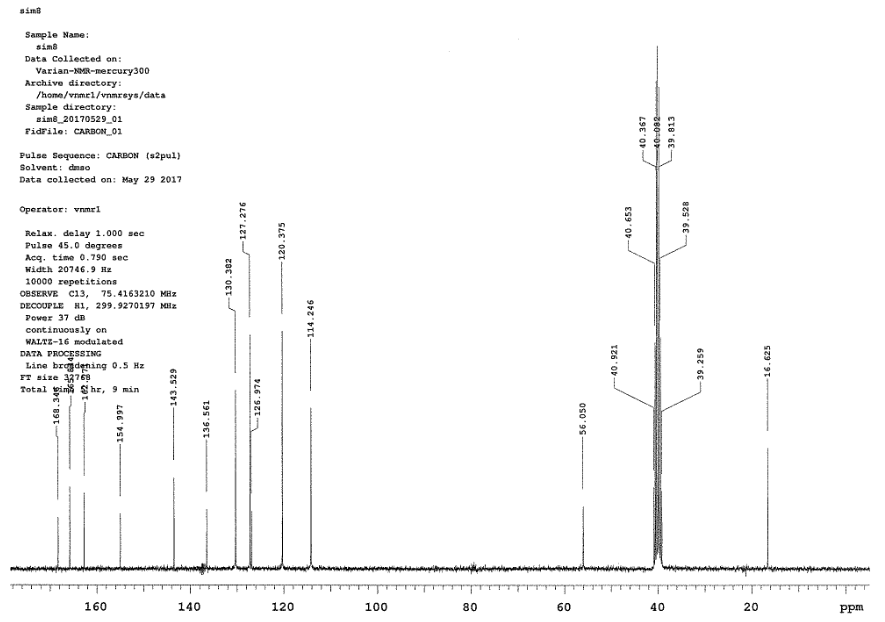


**Figure S19.** ^13^C-NMR spectrum of compound **5**


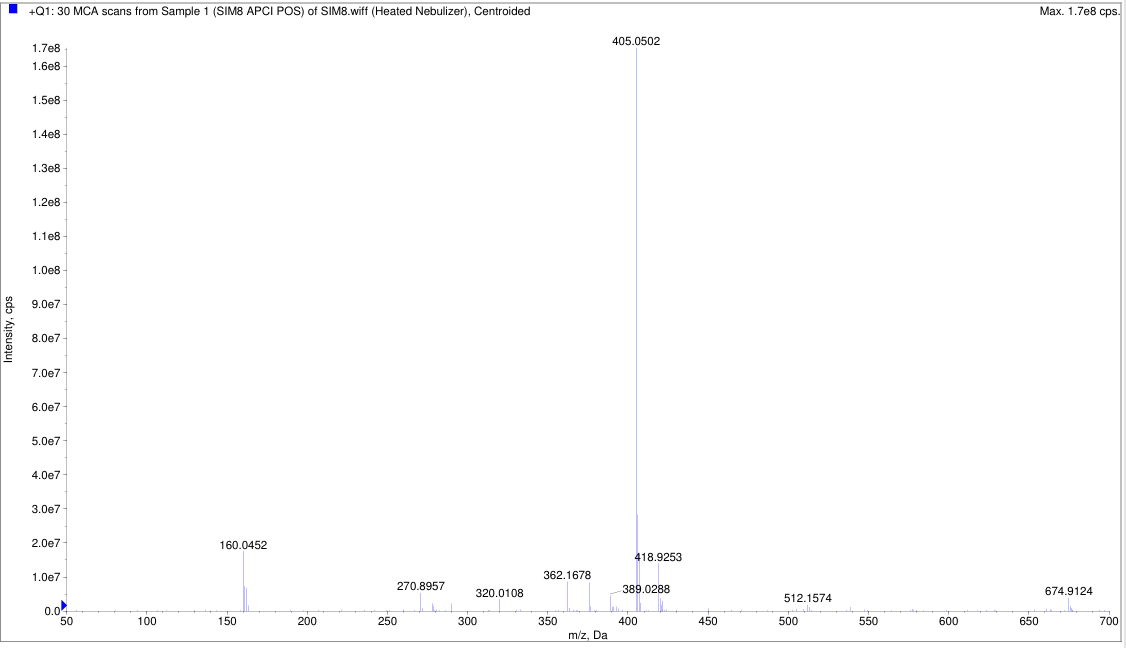


**Figure S20.** Mass spectrum of compound **5**

**
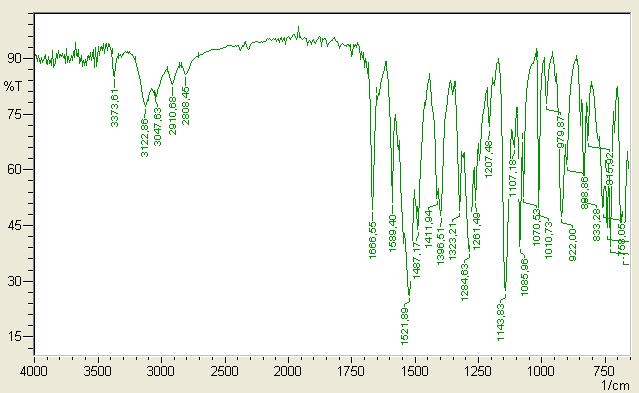
**

**Figure S21.** FTIR spectrum of compound **6**


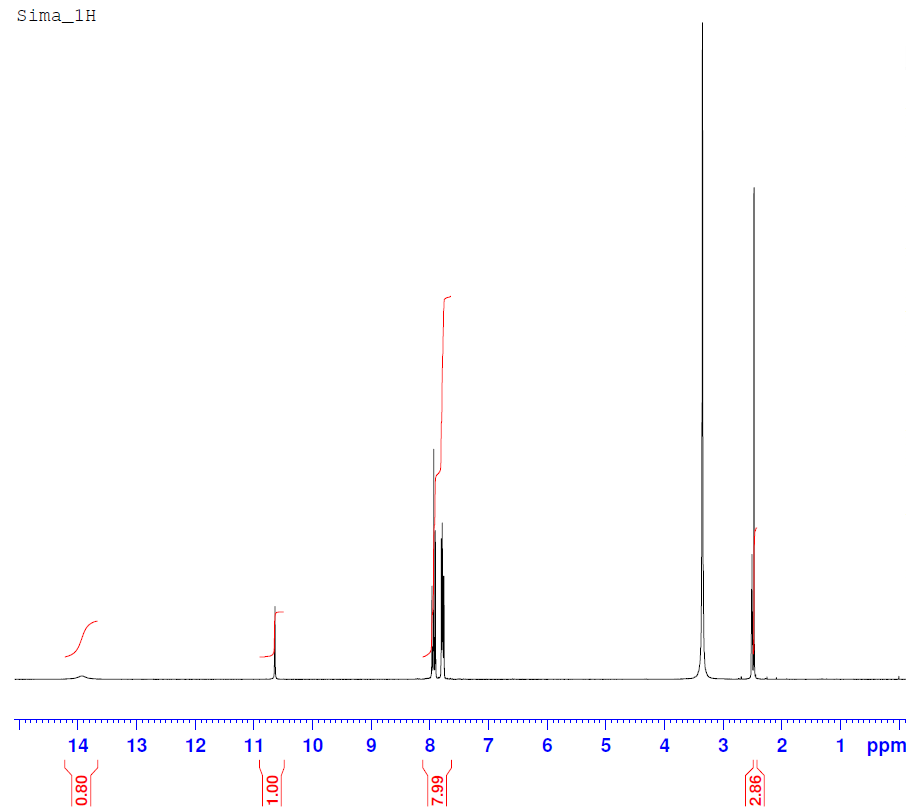


**Figure S22.** ^1^H-NMR spectrum of compound **6**


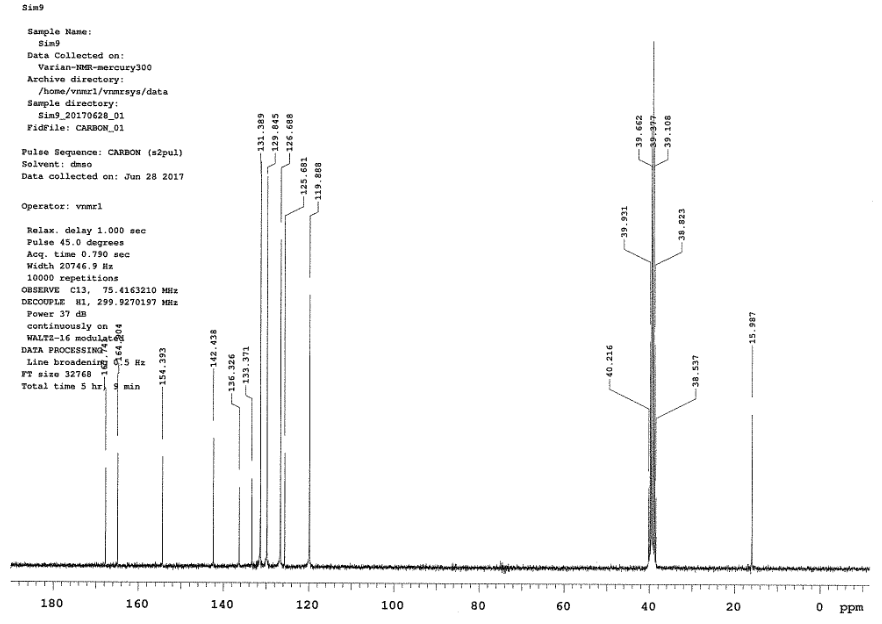


**Figure S23.** ^13^C-NMR spectrum of compound **6**


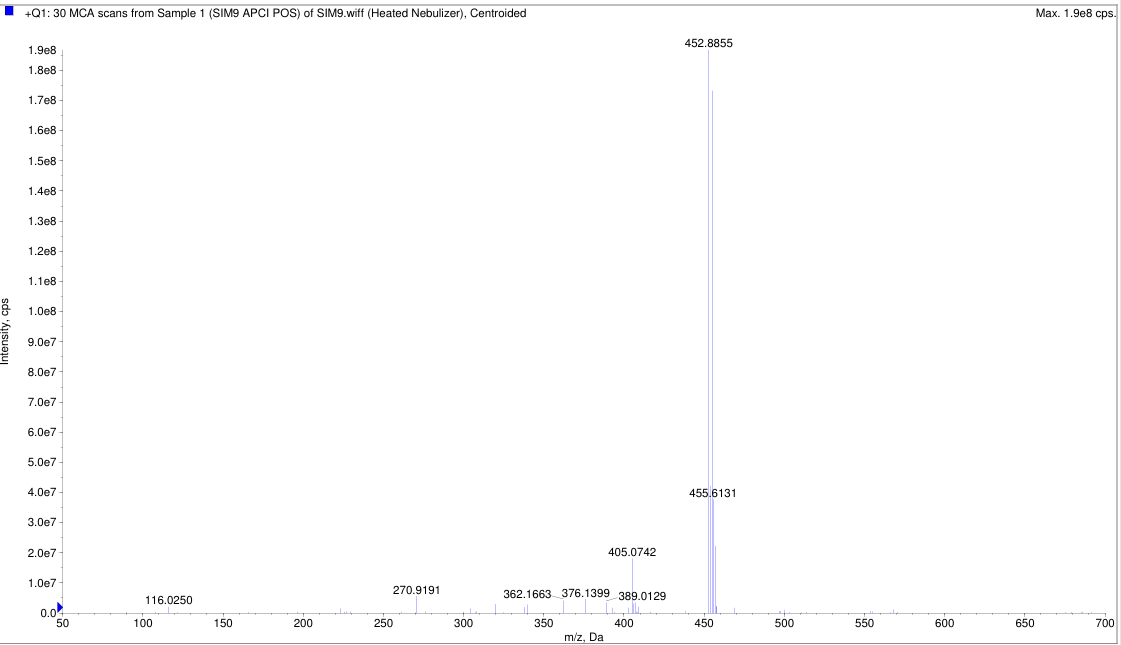


**Figure S24.** Mass spectrum of compound **6**

**
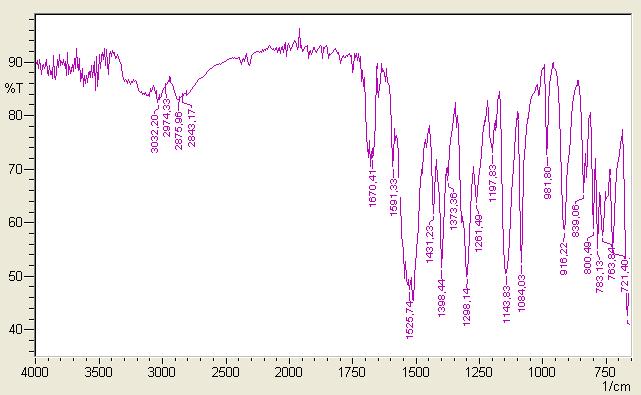
**

**Figure S25.** FTIR spectrum of compound **7**

**
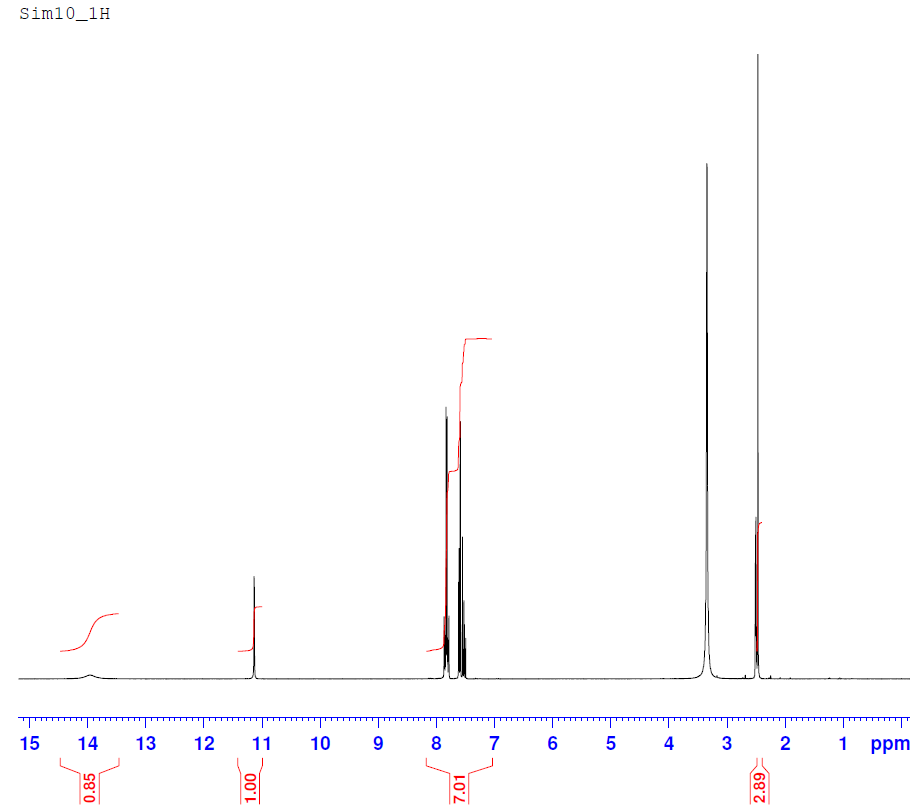
**

**Figure S26.** ^1^H-NMR spectrum of compound **7**


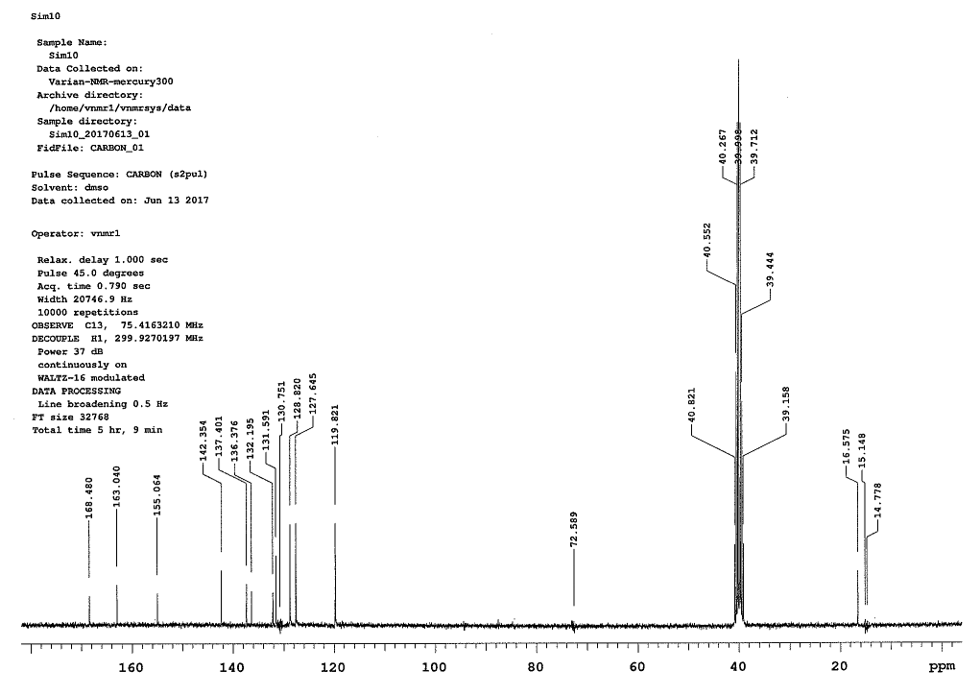


**Figure S27.** ^13^C-NMR spectrum of compound **7**


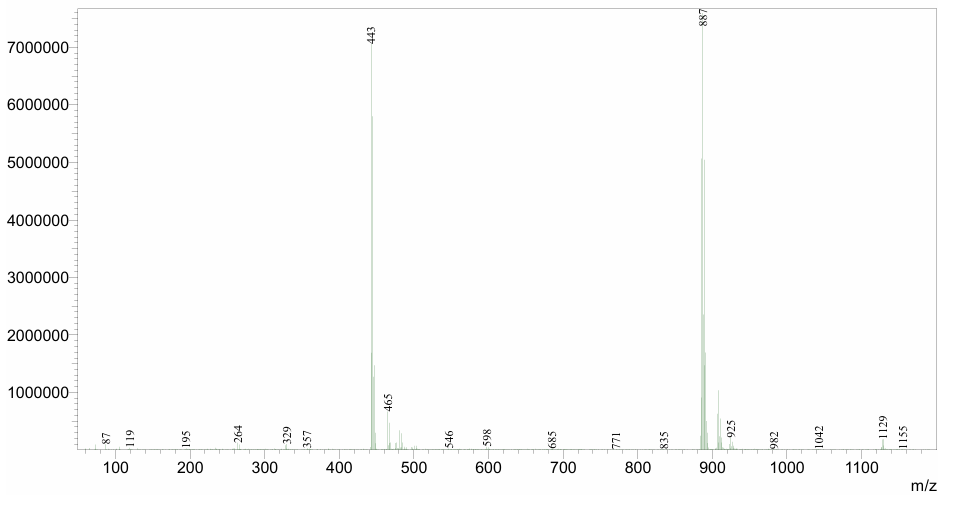


**Figure S28.** Mass spectrum of compound **7**


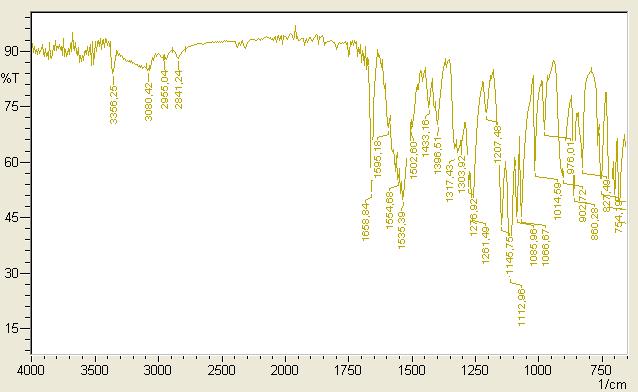


**Figure S29.** FTIR spectrum of compound **8**

**
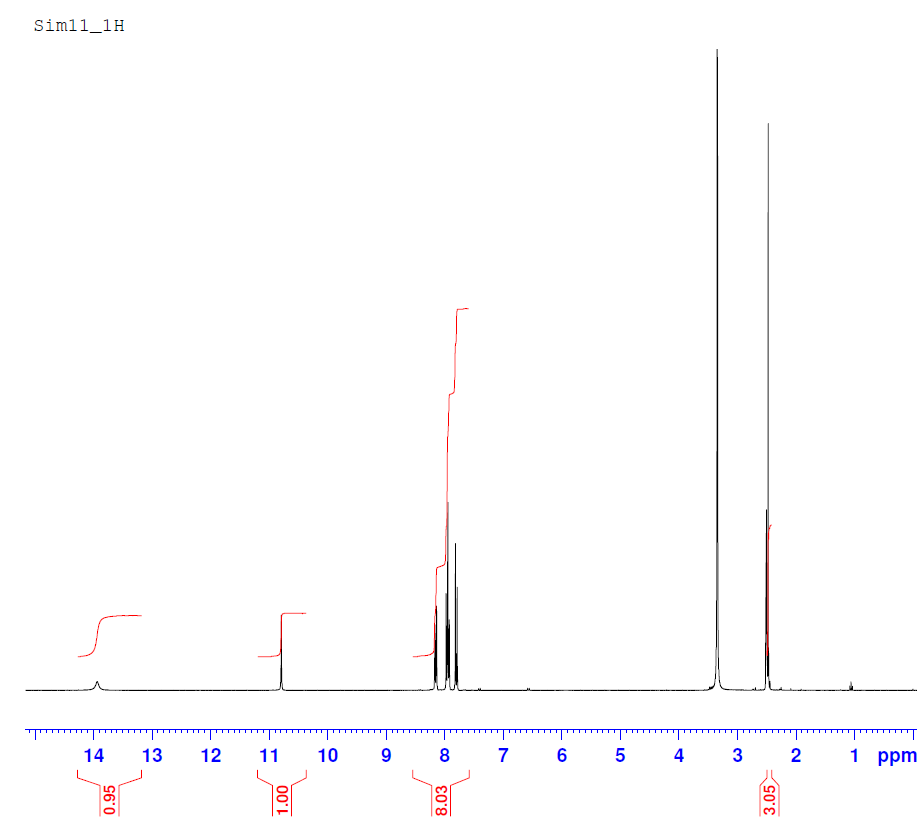
**

**Figure S30.** ^1^H-NMR spectrum of compound **8**


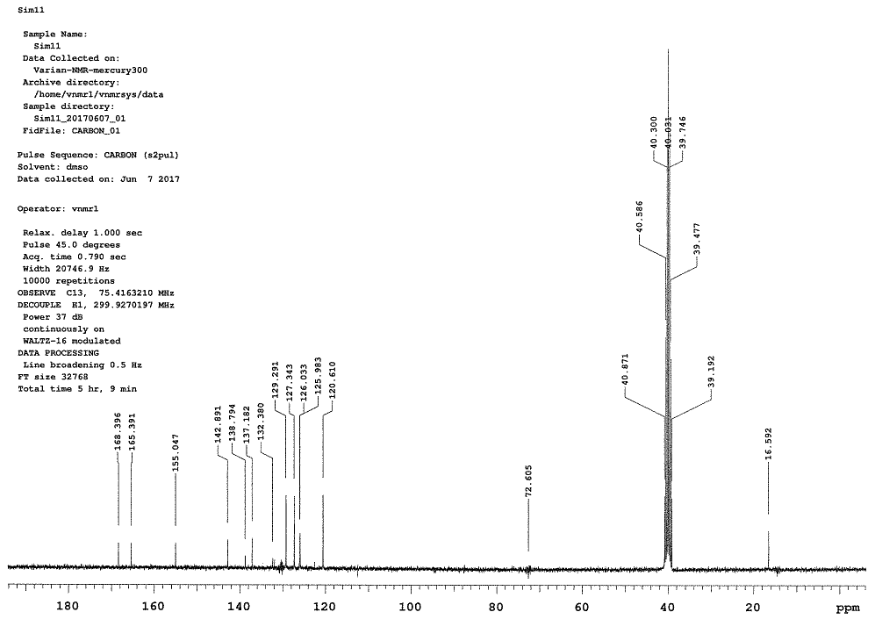


**Figure S31.** ^13^C-NMR spectrum of compound **8**


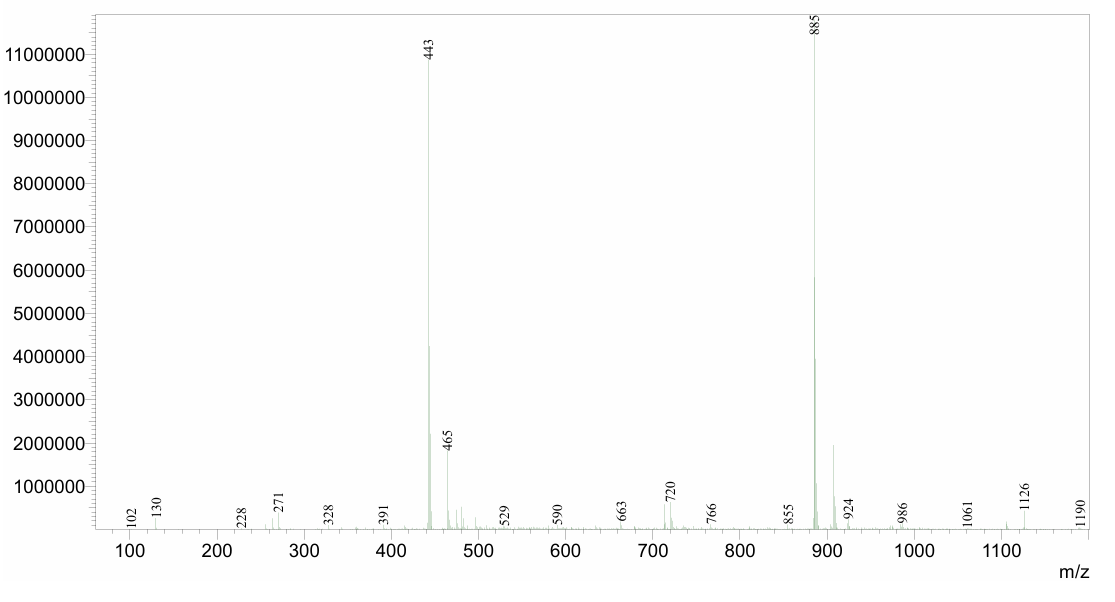


**Figure S32.** Mass spectrum of compound **8**

**
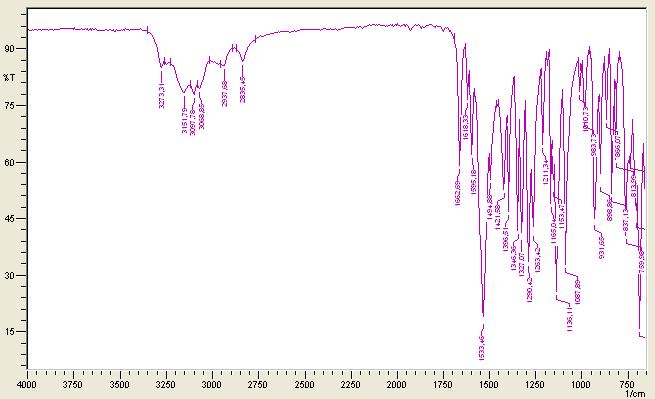
**

**Figure S33.** FTIR spectrum of compound **9**


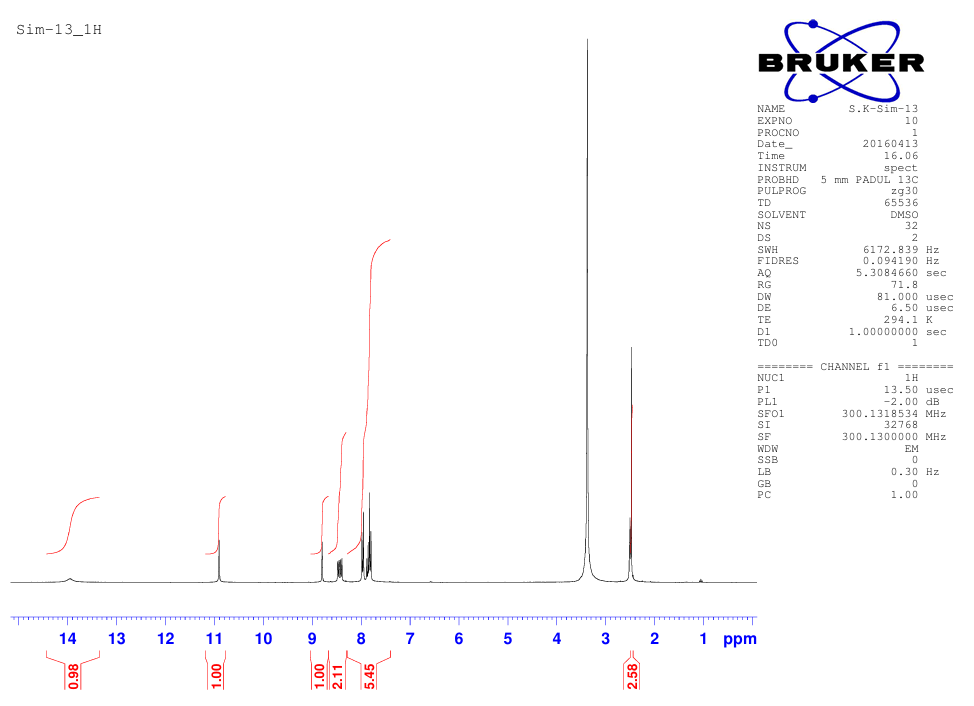


**Figure S34.** ^1^H-NMR spectrum of compound **9**


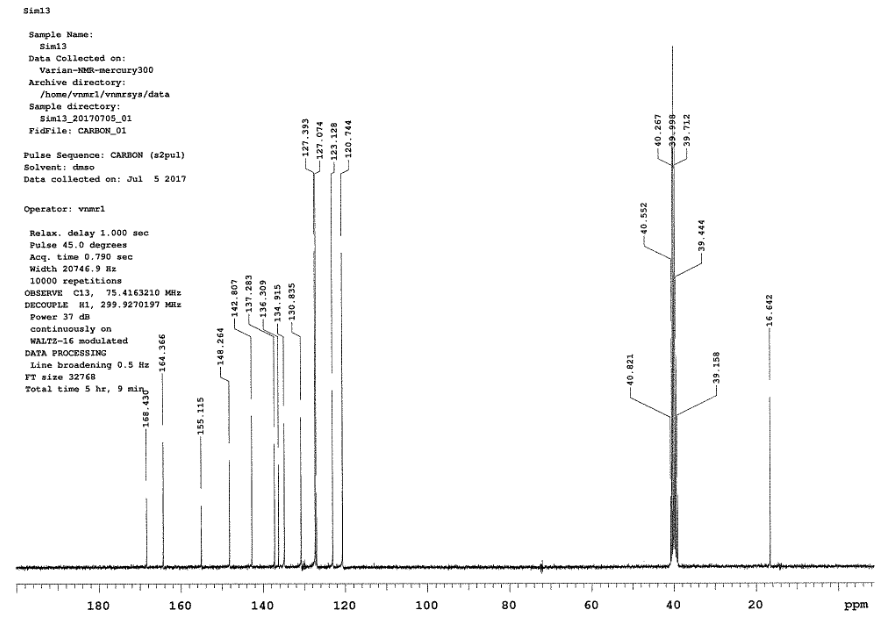


**Figure S35.** ^13^C-NMR spectrum of compound **9**


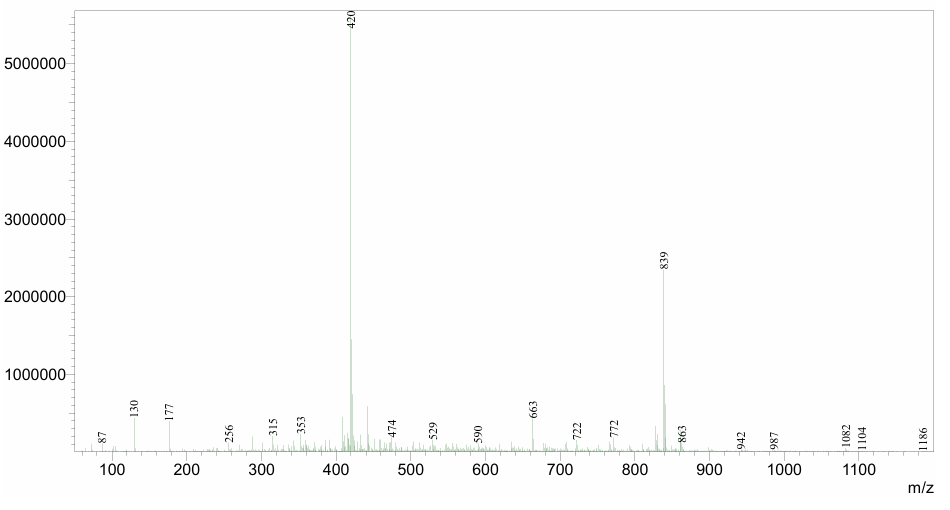


**Figure S36.** Mass spectrum of compound **9**


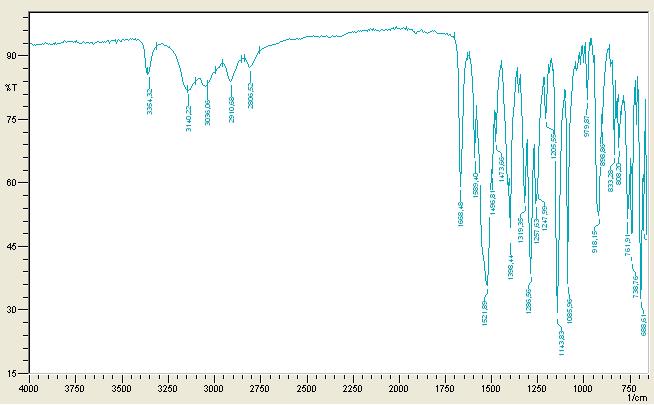


**Figure S37.** FTIR spectrum of compound **10**


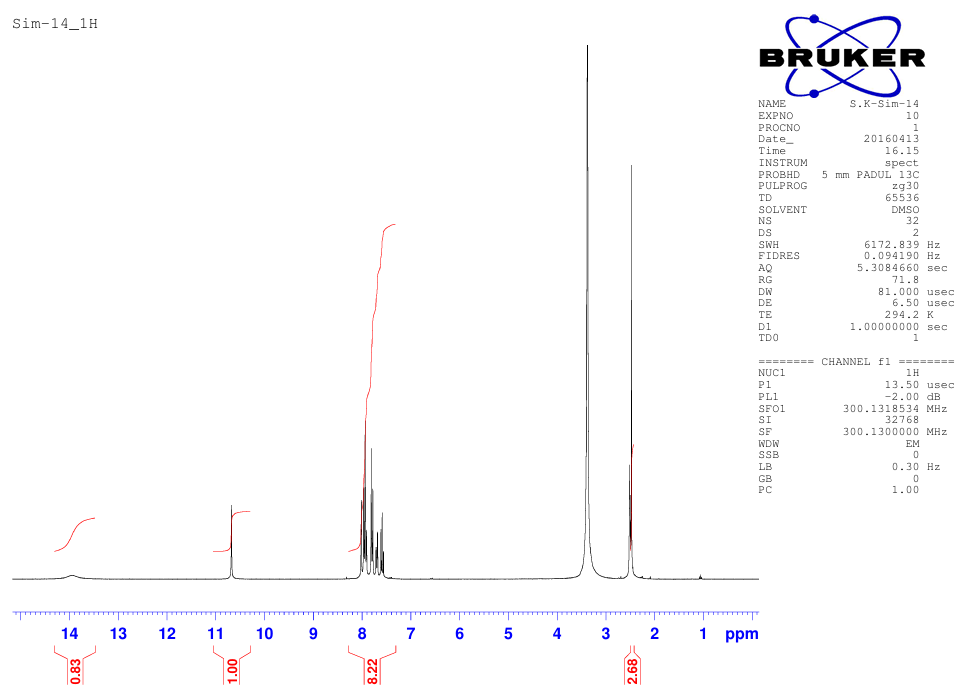


**Figure S38.** ^1^H-NMR spectrum of compound **10**


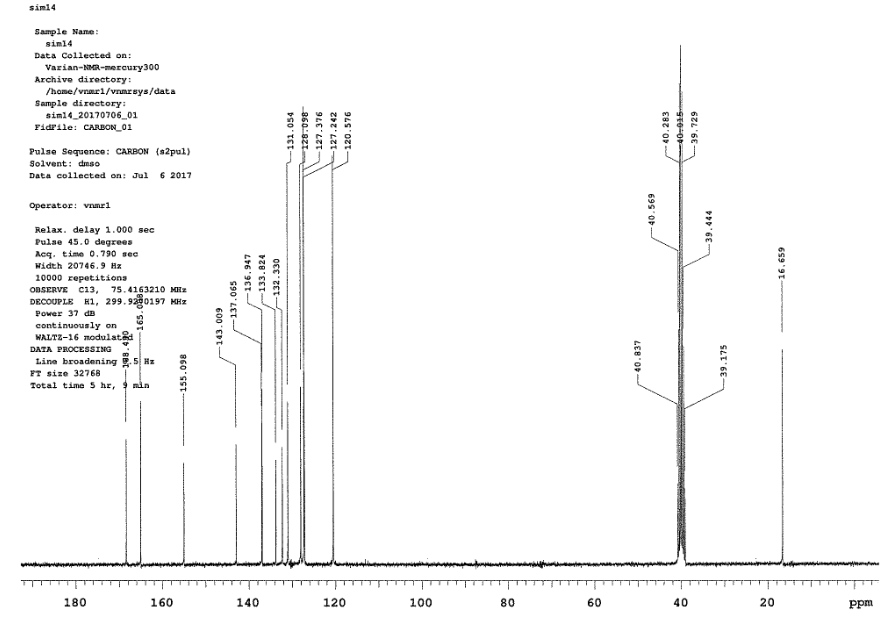


**Figure S39.** ^13^C-NMR spectrum of compound **10**


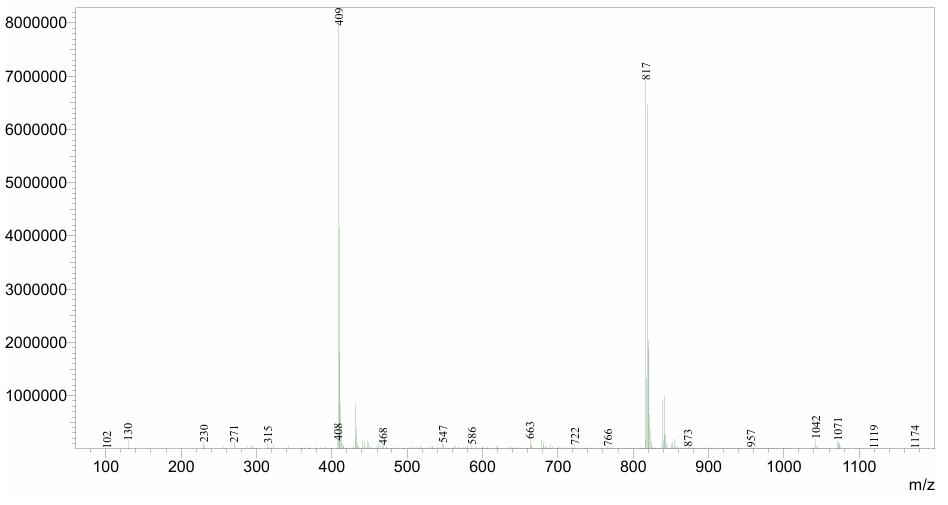


**Figure S40.** Mass spectrum of compound **10**


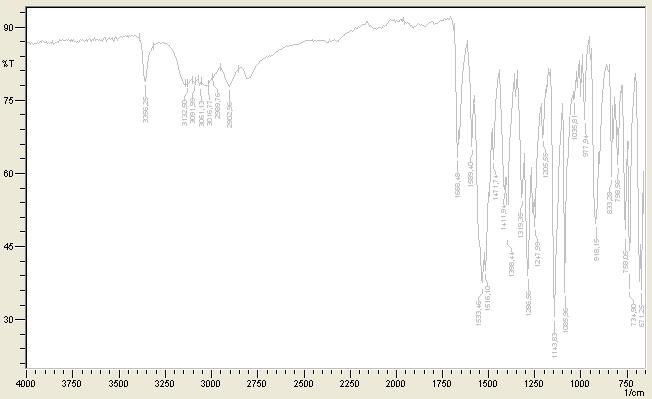


**Figure S41.** FTIR spectrum of compound **11**


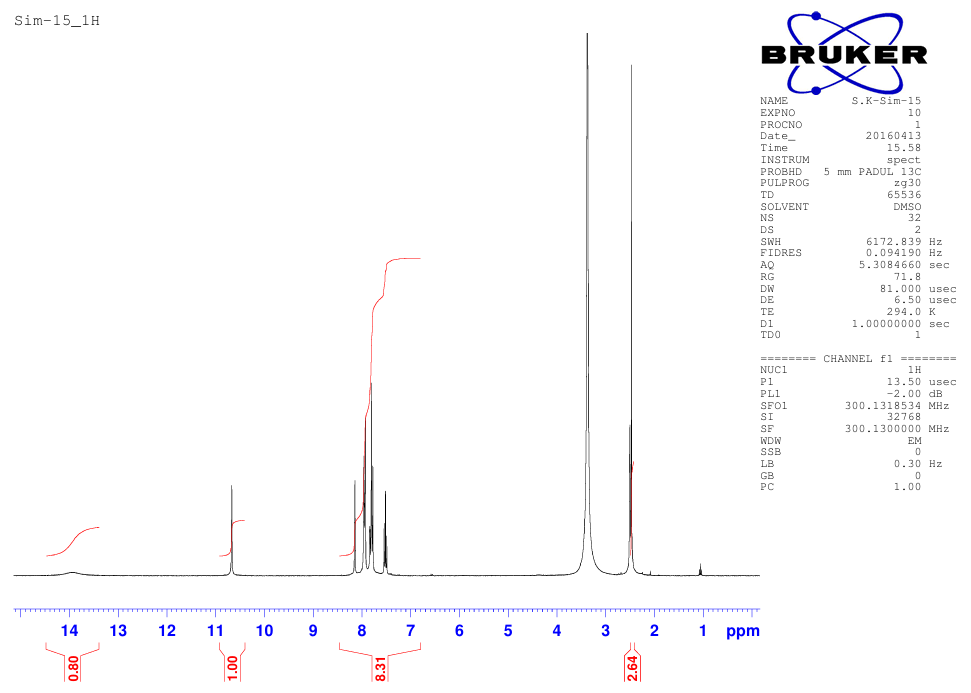


**Figure S42.** ^1^H-NMR spectrum of compound **11**


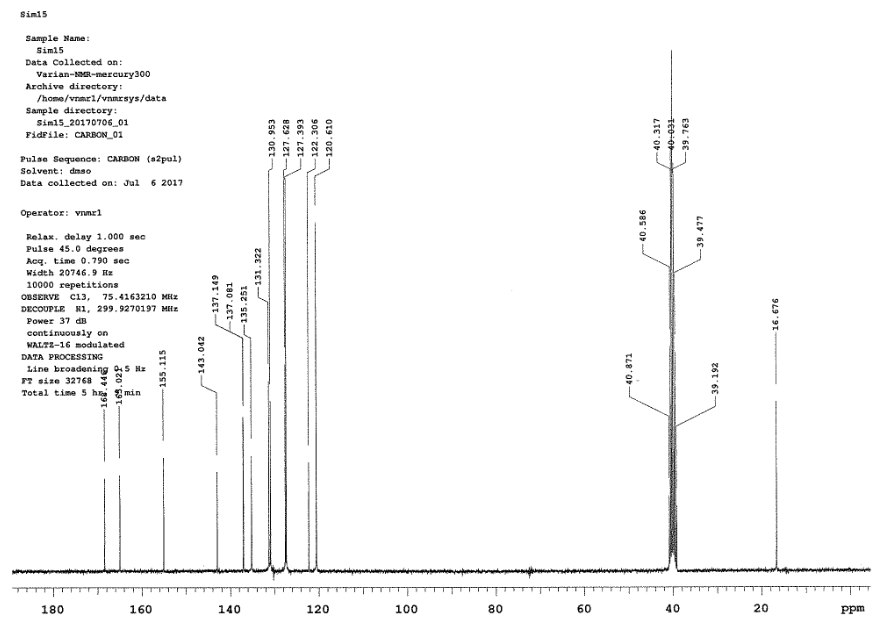


**Figure S43.** ^13^C-NMR spectrum of compound **11**


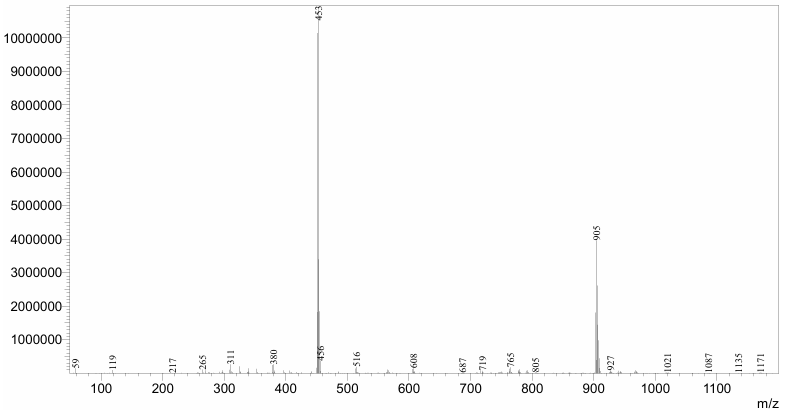


**Figure S44.** Mass spectrum of compound **11**
